# Schema-Informed Digital Mental Health Intervention for Maladaptive Cognitive-Emotional Patterns: Randomized Controlled Trial

**DOI:** 10.2196/65892

**Published:** 2025-08-14

**Authors:** Seohyun Jeong, Hyeonseong Kim, Silvia Kyungjin Lho, Inae Hwang, Seongjun Mun, Soohyun Kim, Hyejin Lim, Hyeonhee Kim, Min-Sup Shin, Woori Moon

**Affiliations:** 1 Department of Psychiatry Seoul National University Hospital Seoul Republic of Korea; 2 40FY Inc Seoul Republic of Korea; 3 Department of Psychiatry Seoul Metropolitan Government-Seoul National University Boramae Medical Center Seoul Republic of Korea; 4 Seoul National University College of Medicine Seoul Republic of Korea

**Keywords:** schema-informed intervention, digital mental health intervention, randomized controlled trial, perfectionism, low self-esteem, social isolation, anxiety

## Abstract

**Background:**

In South Korea, access to mental health services remains limited due to stigma, cost, and self-reliance. While digital interventions can help reduce these barriers, few are designed to address the underlying cognitive-emotional patterns that perpetuate psychological distress.

**Objective:**

This study evaluated the effectiveness of a schema-informed digital intervention (Mindling) aimed at reducing distress and enhancing coping in adults experiencing schema-linked difficulties such as perfectionism (unrelenting standards schema), low self-worth (self-sacrifice schema), social withdrawal (social isolation and alienation schema), and anxiety (negativity and dependence schemas).

**Methods:**

A total of 300 adults (aged 18-60 years) with elevated perceived stress and one dominant distress pattern were recruited on the web and randomized to an intervention group (n=201) or waitlist control (n=99). Participants were assigned to one of four 10-week web-based program modules tailored to their dominant distress pattern: perfectionism (Riggy), low self-worth and fear of rejection (Pleaser), social withdrawal (Shelly), or chronic anxiety (Jumpy). Each module included psychoeducation, guided reflection, and behavioral tasks. Outcomes, including perceived stress, perfectionism, self-esteem, loneliness, and anxiety, were measured at baseline, midintervention (week 5), and postintervention (week 10). Analyses were conducted on 218 participants who completed all 3 assessments (n=138 intervention; n=80 waitlist), using repeated-measures ANOVA or analysis of covariance, as appropriate. In addition, a 1-month follow-up assessment was conducted for the intervention group only to examine maintenance of effects.

**Results:**

Compared with the waitlist group, the intervention group showed significantly greater improvement in perceived stress (*F*_2,432_=27.52; *P*<.001; partial η^2^=0.11) and improvements in pattern-linked outcomes: perfectionism (*F*_2,102_=6.24; *P*=.003; partial η^2^=0.10); self-esteem (*F*_2,96_=11.83; *P*<.001; partial η^2^=0.20); loneliness (*F*_2,110_=8.26; *P*<.001; partial η^2^=0.13); and anxiety (*F*_2,110_=10.75; *P*<.001; partial η^2^=0.16). Secondary outcomes (eg, depression, trait anxiety, and self-efficacy) also improved across most programs. These effects were maintained at a 1-month follow-up. As supplementary findings, adherence was high among those who accessed the platform, with 70.1% (131/187) completing all 10 sessions. The intervention group was also more likely than the waitlist to seek external mental health services (t_216_=2.59; *P*=.01; mean difference=0.45; 95% CI 0.12-0.80).

**Conclusions:**

The schema-informed digital intervention led to significant (*P*<.05) short-term improvements in distress and associated psychological patterns, with high completion rates and increased help-seeking behavior among participants. These findings suggest the potential utility of structured, accessible digital interventions within stepped-care models for mental health support. However, limitations in study design, sample representativeness, and follow-up duration suggest that further research is needed to assess the long-term effectiveness and broader applicability of such digital programs.

**Trial Registration:**

ClinicalTrials.gov NCT06166693; https://clinicaltrials.gov/study/NCT06166693

## Introduction

### Background

While the demand for mental health care is increasing, there is a substantial shortage of professionals who can deliver quality care. Moreover, access to conventional treatments is hindered by the current high costs, long wait times, and social stigma [1,2]. This issue is particularly acute in South Korea [3], as reported by the National Center for Mental Health. In their 2020 survey, only 12.1% of those diagnosed with a mental illness had consulted a mental health professional, which is a strikingly low figure compared with Canada (46.5%), the United States (43.1%), and Australia (34.9%). The substantial disparity between the need and the actual usage of mental health services poses a critical challenge to the mental well-being of the South Korean population, underlining the urgent need to increase engagement in mental health care services.

In the 2020 survey of the National Center for Mental Health, the most common reason (multiple answers allowed) for not using mental health services was the “belief of being able to handle it on one’s own” at 90.3%. Among the 7.2% people who used mental health services over the previous year, 7% visited a psychiatrist. However, access to mental health services in South Korea remains limited, as services are primarily delivered face-to-face within medical institution–based and pose high entry barriers due to psychological and financial concerns. As such, addressing these issues may hold the key to solving the low mental health service use rate. Such psychological and structural barriers point to a notable gap between the increasing demand for mental health services and the availability of timely and appropriate care. Limited access to early intervention may result in the exacerbation of psychological symptoms, delayed recovery, and chronicity of conditions at the individual level, while also increasing long-term health care expenditures and reducing population-level productivity [4].

To address these challenges, non–face-to-face digital interventions have emerged as a promising strategy to improve access to mental health care and reduce treatment delays. Numerous digital mental health platforms have been developed and evaluated across a variety of populations and conditions. For example, IntelliCare, a modular mobile platform combining self-guided tools and coaching, has demonstrated substantial reductions in depression and anxiety symptoms in randomized trials conducted in the United States [5]. Similarly, Woebot, a conversational agent driven by artificial intelligence, has shown promise in improving emotional self-awareness and reducing depressive symptoms, particularly among younger adults [6]. A recent meta-analysis of 39 randomized trials involving nearly 10,000 participants found that both guided and unguided internet-based cognitive behavioral therapy (CBT) were more effective than treatment-as-usual in reducing depressive symptoms, regardless of depression severity [7]. These findings collectively support the efficacy and scalability of digital interventions across diverse clinical contexts.

Building on recent advances in digital mental health, there is growing interest in enhancing intervention effectiveness through personalization. Many digital programs now adapt content based on users’ engagement patterns, self-reported symptoms, or emotional states [8]. While these approaches have improved usability and accessibility, they often prioritize symptom alleviation and diagnostic categories. In contrast, some interventions are beginning to explore how personalization might be based on underlying cognitive-emotional patterns that shape day-to-day functioning.

One such set of tendencies involves stable yet modifiable cognitive-emotional patterns—deep-seated ways of interpreting, feeling, and responding to the world, often rooted in early experiences. These patterns are well captured by the concept of early maladaptive schemas, which refer to enduring self-relevant beliefs formed in response to unmet emotional needs during development [9]. Such patterns can give rise to recurring difficulties such as perfectionism, rejection sensitivity, or emotional inhibition. Because these underlying structures are recognized as contributing to psychological suffering, many therapeutic approaches target them to foster long-term change [10-13].

From a theoretical standpoint, predictive coding models help explain how such change is possible [14]. Early maladaptive schemas can be understood as internal models that guide perception by minimizing prediction error. When individuals encounter experiences that violate their expectations, for example, receiving acceptance when rejection is anticipated, prediction errors may prompt psychological updating. This mechanism underlies many traditional therapies such as CBT, acceptance and commitment therapy (ACT), and schema therapy, which create moments of disconfirmation to shift entrenched beliefs [14].

A recent ecological momentary assessment study further highlights the promise of digital intervention by showing that the activation of these patterns can fluctuate substantially within a single day, depending on situational triggers and emotional states [15]. This contrasts with earlier views that conceptualized these tendencies as static and trait-like, typically requiring intensive, in-person treatment. When these findings are considered alongside predictive coding theory, they suggest that the high accessibility and frequency of digital interventions could provide users with more opportunities to encounter schema-disconfirming experiences in everyday contexts.

Despite this potential, most digital interventions continue to rely on diagnostic categories or symptomatic presentations, with few tailoring content based on users’ deeper psychological profiles. One notable exception is Ong et al [16], who developed a self-guided online program targeting perfectionism through a pattern-based therapy framework. This model emphasizes transdiagnostic processes and flexibly incorporates elements from cognitive, behavioral, and motivational approaches to align with users’ specific difficulties. However, broader applications of such pattern-informed, schema-relevant digital interventions remain scarce.

### Objective

This study aimed to tested a 10-week digital mental health intervention targeting maladaptive cognitive-emotional patterns, such as perfectionism (linked to unrelenting standards schema), low self-esteem (self-sacrifice schema), social withdrawal (social isolation and alienation schema), and anxiety (negativity and dependence schemas), that are closely linked to distress and daily dysfunction. These patterns were not conceptualized as clinical diagnoses or fixed personality traits but as modifiable tendencies shaped by underlying schemas and habitual coping styles. Participants were assigned to 1 of 4 schema-informed intervention tracks based on their dominant distress profile, identified using a brief self-report tool developed specifically for this program. Each module provided targeted content tailored to the user’s primary challenges (eg, fear of failure in perfectionism or rejection sensitivity in low self-esteem), drawing on techniques from schema therapy, cognitive behavioral, mindfulness-based, and behavioral activation therapies. The intervention focused on promoting adaptive cognitive interpretations, emotional responses, and behavioral coping strategies aligned with each participant’s dominant pattern, rather than attempting to alter fundamental personality structures. Ultimately, this study aimed to determine whether meaningfully personalized, scalable digital intervention could effectively address psychological distress and enhance daily functioning.

## Methods

### Study Design and Participants

#### Overview

A randomized controlled trial was conducted with a 201-person intervention group versus a 99-person waitlist group to test the effectiveness of a digital intervention program on maladaptive cognitive-emotional patterns linked to early schemas and real-world distress. Screening, informed consent, allocation to conditions, assessment, and delivery of the intervention were conducted online. In addition, detailed explanations of the study and instructions on how to use the research platform were provided via phone calls and email.

#### Initial Screening

Participants were recruited from November 23, 2022, to January 6, 2023, through an online community for university students, office workers, and homemakers in South Korea. Adults aged 18 to 60 years were eligible to participate if, at screening, they reported at least a moderate level of perceived stress (10-item Perceived Stress Scale [PSS-10] ≥17) along with elevated levels of 1 or more maladaptive cognitive-emotional patterns: perfectionism (Hewitt Multidimensional Perfectionism Scale [HMPS] ≥198), low self-esteem (State Self-Esteem Scale [SSES] ≤57), loneliness (University of California, Los Angeles Loneliness Scale ≥47), or anxiety (Beck Anxiety Inventory [BAI] ≥16). The cutoff scores for each scale were determined a priori, based on the upper or lower 25% of score distributions observed in a separate dataset (N=250) collected during a previous validation study of the Maladaptive Personality and Interpersonal Schema Scale [17]. These thresholds were established to identify individuals with meaningful levels of schema-linked psychological difficulties corresponding to the target domain of each intervention module, thereby improving the relevance of participant-program matching. When participants met multiple trait-based criteria, program assignment was determined using the domain associated with the highest standardized (*z* scored) value.

#### Allocation to Condition

On the basis of the screening results, 75 participants were assigned to each of the 4 intervention programs. After final confirmation of consent to participate in the study, participants were randomly assigned to the intervention group (n=201) or the waitlist control group (n=99) using a 2:1 allocation ratio. This ratio was predetermined to ensure adequate sample sizes within each of the 4 intervention modules and to accommodate potential attrition in the intervention group. Randomization was performed using a computer-generated allocation list created. The assignment was conducted by an independent researcher, and the research assistant who enrolled participants was blinded to group allocation, study hypotheses, and outcome assessments. Although the planned sample size was 200 for the intervention group and 100 for the waitlist group, 1 additional participant was assigned to the intervention group during the randomization process, resulting in final group sizes of 201 and 99, respectively.

**Figure 1:**
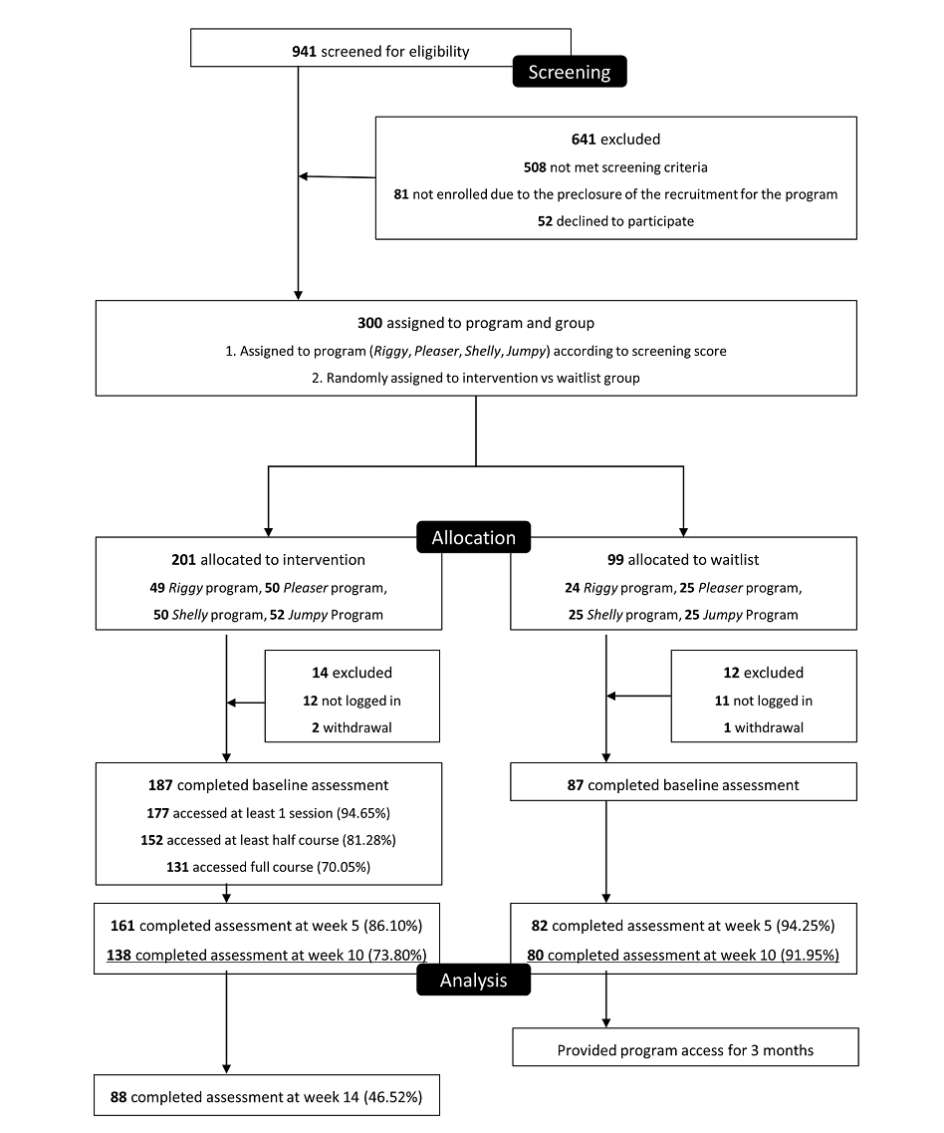
Flow diagram of participant recruitment, allocation, follow-up, and analysis.

Of the 300 participants initially randomized, 23 did not log in to the web-based platform, and therefore did not initiate any part of the study. An additional 3 participants logged in but withdrew their consent before completing the baseline assessment. These 26 participants did not proceed with the study procedures, resulting in 187 participants in the intervention group and 87 in the waitlist control group (Figure 1). As randomization occurred before participant engagement, these exclusions were unlikely to introduce systematic bias. The intervention group followed the assigned digital program for 10 weeks, while the waitlist group remained inactive during the same period.

### Intervention

#### Intervention Delivery and Participant Management

The Mindling intervention was delivered via a dedicated web-based platform accessible through web browsers and mobile apps. On the basis of schema therapy, it targeted maladaptive cognitive-emotional patterns—perfectionism, low self-esteem with fear of rejection, social withdrawal, and anxiety. Therapeutic principles from schema therapy, CBT, ACT, behavioral therapy, mindfulness-based cognitive therapy (MBCT), and positive psychology were integrated for self-guided online delivery. Participants were assigned to 1 of 4 modules (Riggy, Pleaser, Shelly, or Jumpy) based on a brief self-assessment [17].

Each program consisted of 10 weekly sessions (40-60 min each), structured into cognitive, emotional, and behavioral subsections. Automated texts reminded participants to engage regularly, and additional follow-up occurred if participation lagged by more than 2 weeks. Waitlist participants gained program access after completing the final assessment.

#### Program Structure

The intervention was structured to address maladaptive cognitive-emotional patterns across 3 temporal perspectives—past, present, and future—offering a coherent therapeutic flow throughout the 10-week program. Each perspective corresponded to a distinct therapeutic focus. In the past perspective (schema therapy), participants reflected on the developmental origins of their schemas, validated past emotional experiences, and identified core beliefs and underlying strengths through narrative exercises and guided imagery. In the present perspective (CBT and MBCT), current maladaptive thoughts and behaviors were addressed through structured modules focusing on cognitive restructuring, mindfulness practices, emotional regulation, and behavior modification. In the future perspective (ACT and positive psychology), participants explored personal values, clarified long-term goals, and planned value-based actions that leveraged their identified strengths for sustained psychological growth.

Content across these modules was tailored to each participant’s dominant pattern, enhancing relevance and engagement.

#### Program-Specific Modules

Each of the 4 programs was categorized based on schema therapy and linked to specific maladaptive schemas. Module content also incorporated techniques from schema therapy, CBT, MBCT, ACT, behavioral therapy, and positive psychology with variation in emphasis depending on the dominant psychological pattern: Riggy (perfectionism, linked to the schema domain of overvigilance and inhibition, specifically the unrelenting standards schema) focused on perfectionism-related beliefs, rigid thinking patterns, and adaptive strategies for managing performance pressure. Pleaser (low self-esteem, linked to the schema domain of other-directedness, specifically the self-sacrifice schema) addressed rejection sensitivity, self-neglect, and interpersonal assertiveness. Shelly (social withdrawal, linked to the schema domain of disconnection and rejection, specifically the social isolation schema) targeted avoidance behaviors, negative self-image, and gradual social re-engagement. Jumpy (anxiety, linked to the schema domains of impaired autonomy and performance through the dependence schema, and overvigilance and inhibition through the negativity and pessimism schema) focused on anxiety management, reduction of cognitive distortions, and exposure-based coping techniques.

To support engagement, the program incorporated interactive digital worksheets, self-assessments, psychoeducational content, mindfulness audio, and visual prompts. Symbolic program characters and a guiding therapist figure (Minder) enhanced emotional involvement and reflection, promoting adaptive coping strategies without direct therapist interaction.

### Assessment

The assessment schedule was as follows: (1) preintervention (baseline) at week 0; (2) mid-intervention at week 5; (3) postintervention at end of intervention (week 10), and (4) follow-up 4 weeks after intervention (week 14). The intervention group was assessed at weeks 0, 5, 10, and 14, and the waitlist group at weeks 0, 5, and 10. All assessments included 1 primary outcome measure depending on the program to which the participants were assigned and 5 additional scales measuring stress levels, self-efficacy, depression, and anxiety. Each participant was paid KRW 10,000 (US $7.76) per assessment after completing all study procedures.

### Study Outcomes

#### Primary Outcomes

The primary outcome for the overall intervention was the level of perceived stress, which served as a common indicator across all intervention programs. Each program focused on a specific schema-linked difficulty and corresponding outcome: perfectionism for unrelenting standards in the Riggy program, self-esteem for self-sacrifice in the Pleaser program, loneliness for social isolation and alienation in the Shelly program, and anxiety for negativity and dependence schemas in the Jumpy program. Other psychological constructs such as depression, trait anxiety, and self-efficacy were assessed as secondary outcomes.

Perceived stress, the primary outcome for the overall Mindling program was measured using the 10-item PSS-10 [18]. The total score ranged from 0 to 40, with a higher total score indicating a higher level of stress. The internal consistency (Cronbach α at baseline) in this study was 0.79.

The primary outcome of the Riggy program was perfectionism, which reflects distress associated with the unrelenting standards schema, and was measured using the HMPS [19]. The HMPS consists of 3 subscales: “self-oriented perfectionism,” “other-oriented perfectionism,” and “socially prescribed perfectionism.” There are 45 items in total, with the total score ranging from 45 to 315, a higher total score indicating a higher level of perfectionism. In this study, the internal consistency (Cronbach α at baseline) was 0.86 for the overall scale.

The primary outcome of the Pleaser program was state self-esteem, reflecting distress linked to the self-sacrifice schema in the other-directedness domain, and was measured using the SSES [20]. The SSES comprises “performance self-esteem,” “appearance self-esteem,” and “social self-esteem.” There are 20 items in total, with the total score ranging from 20 to 100, a higher total score indicating a higher level of state self-esteem. In this study, the internal consistency (Cronbach α at baseline) was 0.85 for the overall scale.

The primary outcome of the Shelly program was loneliness, reflecting distress related to the social isolation and alienation schema in the disconnection and rejection domain, and was measured using the UCLA Loneliness Scale [21,22]. There are 20 items in total, with the total score ranging from 0 to 80, and a higher total score indicating a higher level of loneliness. The internal consistency (Cronbach α at baseline) in this study was 0.89.

The primary outcome of the Jumpy program was anxiety, reflecting distress linked to the negativity and dependence schemas within the overvigilance and impaired autonomy domains, and was measured using the BAI. The primary measure of the Jumpy program was the BAI [23], which consists of subfactors including “subjective anxiety,” “neurophysiological anxiety,” “autonomic nervous system anxiety,” and “panic anxiety.” There are 21 items in total, with the total score ranging from 0 to 63; a total score of 0 to 7 indicates normal anxiety, 8 to 15 indicates mild anxiety, 16 to 25 indicates moderate anxiety, and 26 to 63 indicates severe anxiety. In this study, the internal consistency (Cronbach α at baseline) was 0.91 for the overall scale.

#### Secondary Outcomes

Exploratory secondary efficacy data were collected from all participants. Self-efficacy was measured using the Self-Efficacy Scale (Cronbach α in this study=0.92) [24]. The level of depression was measured using the Center for Epidemiologic Studies Depression Scale (Cronbach α in this study=0.91) [25], and the level of anxiety was measured using the trait anxiety scale of State-Trait Anxiety Inventory (Cronbach α in this study=0.94) [26]. In addition, the level of stress, as measured by the PSS-10, was used as a secondary efficacy measure for each program.

#### Sociodemographic and Mental Health Service Use Information

Demographic information regarding age and gender was collected during the initial screening phase, with additional information on education level, marital status, and occupation during the baseline assessment. Mental health service use was considered a potential covariate and was identified in all assessments by collecting information on the use of medication, psychotherapy, outpatient services, and other digital mental health interventions. On the basis of this, the variables were configured as “current mental health service” and “current prescription medication,” dichotomized into “yes” or “no” for mental health service use and prescription medication.

### Statistical Analysis

A power analysis was conducted using G*Power (version 3.1.9.7; Heinrich Heine University Düsseldorf) for a repeated-measures ANOVA within-between interaction. An effect size of 0.4 was selected based on previous digital intervention studies that reported moderate to large effects [27,28]. To achieve 80% power (α=0.05), which is a commonly accepted threshold for behavioral intervention research, a minimum of 52 participants per intervention module was required. Considering a potential dropout rate of up to 50% in digital mental health interventions [29], the target enrollment was set at 200 for the intervention group (50×4 modules) and 100 for the waitlist control group (25×4 modules), following a 2:1 allocation ratio.

Analyses were conducted on a per-protocol basis, including only participants who completed all 3 assessment time points (baseline, postintervention, and follow-up). This approach was chosen to evaluate the effectiveness of the intervention among participants who fully engaged with and completed the program, aligning with the study’s goal of assessing real-world usage effects. All analyses were performed separately for the total sample and for each individual program (Riggy, Pleaser, Shelly, and Jumpy), with the same procedures conducted 5 times.

Repeated-measures ANOVA and analyses of covariance (ANCOVA) were used to evaluate changes in psychological outcomes over time within and between groups. These methods are well suited to longitudinal data with multiple assessment points, as they account for within-subject correlations and allow for the analysis of time×group interactions. ANCOVA was applied when baseline group differences were identified in demographic or clinical characteristics, with such variables entered as covariates to improve the precision of effect estimation. All *t* tests were 2-tailed, and the significance threshold was set at α=.05. The statistical procedures used to evaluate intervention effects, conducted using SPSS for Windows (version 20.0; IBM Corp), proceeded as follows: (1), independent samples *t* tests were used for continuous variables, and chi-square analyses for categorical variables, to assess baseline equivalence between the intervention and waitlist groups; (2), repeated-measures ANOVA or ANCOVA was conducted to examine group-by-time interaction effects; (3), if a significant interaction was found, post hoc univariate ANOVAs or ANCOVAs were performed at each assessment point; (4), additional analyses examined whether intervention effects remained significant after controlling for mental health service or medication use. A total of 2 composite covariates (ranging from 0-3) were constructed by summing the number of times participants reported use of mental health services or medications at weeks 0, 5, and 10. These were entered into repeated-measures ANCOVA for further adjustment, but only in cases where baseline group differences on these covariates were present; and (5), paired *t* tests were conducted within the intervention group to evaluate whether effects persisted from postintervention to follow-up.

### Ethical Considerations

This study was approved by the institutional review board (IRB) of the Seoul National University Hospital (2203-108-1309) and registered at ClinicalTrials.gov (NCT06166693). All participants provided informed consent on the web before enrollment and were informed of their right to withdraw at any time. All collected data were anonymized before analysis to ensure confidentiality. Participants received a compensation of KRW 10,000 (approximately US $7.76) per assessment. The study was conducted in accordance with the approved IRB protocol, and no major deviations occurred during implementation.

## Results

### Sample Characteristics and Intervention Adherence

Of the 300 participants who were randomized after screening, 201 were assigned to the intervention group and 99 to the waitlist group. A total of 218 participants, 138 in the intervention group and 80 in the waitlist group, completed all 3 assessment time points (pre-, mid-, and postintervention) and were included in the final per-protocol analysis (Figure 1). The completion rate was higher in the waitlist group (80/99, 92%) than in the intervention group (138/187, 73.8%).

Adherence to the intervention was tracked automatically through log data collected by the Mindling platform, which recorded session access and completion. Among the 187 intervention participants who logged into the platform and did not withdraw before the baseline assessment, 177 (94.7%) engaged in at least 1 session. Furthermore, 152 participants (81.3%) completed 5 or more sessions, and 131 (70.1%) completed all 10 sessions. These adherence rates are reported independently from the per-protocol analysis set.

The mean age of the analytic sample was 31.95 (SD 8.09; range 18-58) years, and 84.4% (184/218) were female. Approximately 26.6% (58/218) were married, and participants had an average of 15.27 (SD 2.11; range 12-20) years of education. In addition, 80.3% (175/218) were either employed or enrolled in school at baseline. At baseline, 30.3% (66/218) of participants reported current use of mental health services, and 21.6% (47/218) were taking prescription psychiatric medications. To better contextualize group characteristics, demographic and clinical features by assigned program (Riggy, Pleaser, Shelly, and Jumpy) are shown in Table 1.

**Table 1:** Demographic and clinical features by assigned program.

Program	Age (years), mean (SD; range)	Female participants, n/N (%)	Married, n/N (%)	Education (years), mean (SD; range)	Employed/in school, n/N (%)	Current mental health service use, n/N (%)	Prescription medication use, n/N (%)
Total	31.95 (8.09; 18–58)	184/218 (84)	58/218 (27)	15.27 (2.11; 12–20)	175/218 (80)	66/218 (30)	47/218 (22)
Riggy	34.76 (8.98; 19-54)	45/54 (83)	26/54 (48)	16.22 (2.04; 12-20)	49/54 (90)	10/54 (19)	8/54 (15)
Pleaser	29.74 (7.07; 18-48)	39/50 (78)	5/50 (10)	14.64 (2.08; 12-18)	35/50 (70)	15/50 (30)	10/50 (20)
Shelly	32.00 (8.75; 19-58)	49/57 (86)	12/57 (21)	15.26 (2.13; 12-20)	42/57 (74)	20/57 (35)	13/57 (23)
Jumpy	31.19 (6.66; 20-45)	51/57 (89)	15/57 (26)	14.91 (1.93; 12-18)	49/57 (86)	21/57 (37)	16/57 (28)

### Differences by Intervention Condition

At baseline, there were no significant differences in demographic characteristics between the intervention and waitlist group across the total, Pleaser, Shelly, and Jumpy programs (Table 2). However, in the Riggy program, participants in the intervention group were significantly older than those in the waitlist group, t_52_=2.62; *P*=.01; mean difference=6.29 y; 95% CI 1.47-11.10. There were no significant between-group differences at baseline for the primary outcomes: perceived stress in the total program (t_216_=−1.07; *P*=.29), perfectionism in the Riggy program (t_52_=−0.57; *P*=.57), self-esteem in the Pleaser program (t_48_=−0.45; *P*=.63), loneliness in the Shelly program (t_55_=−1.45; *P*=.15), and anxiety in the Jumpy program (t_55_=0.93; *P*=.36). No significant group differences were observed for any secondary outcomes at baseline (see Table S1 in Multimedia Appendix 1 for full statistics).

**Table 2:** Preintervention (baseline) characteristics of participants.

		Intervention	Waitlist	*t* or chi-square test (*df*)	*P* value
**Total program**					
	Sample size, n	138	80	—^a^	—
	Age (y), mean (SD)	32.62 (8.16)	30.80 (7.89)	1.61 (216)	.11
	Female, n (%)	119 (87.23)	65 (81.25)	0.96 (1)	.33
	Married, n (%)	39 (28.26)	19 (23.75)	0.62 (1)	.43
	Education (y), mean (SD)	15.43 (2.19)	14.98 (1.96)	1.55 (216)	.12
	Student or employed, n (%)	113 (81.88)	62 (77.50)	0.62 (1)	.43
	Current mental health services, n (%)	47 (34.06)	19 (23.75)	2.55 (1)	.11
	Current prescription medications, n (%)	33 (23.91)	14 (17.50)	1.23 (1)	.27
**Riggy program**					
	Sample size, n	34	20	—	—
	Age (y), mean (SD)	37.09 (8.78)	30.80 (8.04)	2.62 (52)	.01
	Female, n (%)	29 (85.29)	16 (80)	0.25 (1)	.71
	Married, n (%)	18 (52.94)	8 (40)	0.85 (1)	.36
	Education (y), mean (SD)	16.59 (2.12)	15.60 (1.79)	1.75 (52)	.09
	Student or employed, n (%)	33 (97.06)	16 (80)	4.36 (1)	.06
	Current mental health services, n (%)	7 (20.59)	3 (15)	0.26 (1)	.73
	Current prescription medications, n (%)	6 (17.65)	2 (10)	0.58	.70
**Pleaser program**					
	Sample size, n	31	19	—	—
	Age (y), mean (SD)	30.16 (7.53)	29.05 (6.40)	0.53 (48)	.60
	Female, n (%)	24 (77.42)	15 (78.95)	0.02 (1)	.90
	Married, n (%)	5 (16.13)	0 (0)	3.41 (1)	.14
	Education (y), mean (SD)	14.90 (2.12)	14.21 (1.99)	1.15 (48)	.26
	Student or employed, n (%)	24 (77.42)	11 (57.89)	2.14 (1)	.14
	Current mental health services, n (%)	11 (35.48)	4 (21.05)	1.17 (1)	.28
	Current prescription medications, n (%)	7 (22.58)	3 (15.79)	0.34	.72
**Shelly program**					
	Sample size, n	35	22	—	—
	Age (y), mean (SD)	32.17 (8.22)	31.73 (9.75)	0.19 (55)	.85
	Female, n (%)	30 (85.71)	19 (86.36)	0.01 (1)	.99
	Married, n (%)	7 (20)	5 (22.72)	0.10 (1)	.99
	Education (y), mean (SD)	15.43 (2.15)	15.00 (2.12)	0.74 (55)	.46
	Student or employed, n (%)	24 (68.57)	18 (81.82)	1.22 (1)	.27
	Current mental health services, n (%)	12 (34.29)	8 (36.36)	0.03 (1)	.87
	Current prescription medications, n (%)	7 (20)	6 (27.27)	0.41 (1)	.52
**Jumpy program**					
	Sample size, n	38	19	—	—
	Age (y), mean (SD)	31.05 (6.60)	31.47 (6.95)	-0.22 (55)	.82
	Female, n (%)	36 (94.74)	15 (78.95)	3.35 (1)	.09
	Married, n (%)	9 (23.68)	6 (31.58)	0.41 (1)	.52
	Education (y), mean (SD)	14.84 (2.01)	15.05 (1.81)	-0.39 (55)	.70
	Student or employed, n (%)	32 (84.21)	17 (89.47)	0.29 (1)	.71
	Current mental health services, n (%)	17 (44.74)	4 (21.05)	3.05 (1)	.08
	Current prescription medications, n (%)	13 (34.21)	3 (15.79)	2.13 (1)	.15

^a^Not applicable.

Across all programs, mental health service use and psychiatric medication rates did not differ between groups at the preintervention assessment (Table 2). However, a significant increase in mental health service use was observed in the intervention group between the pre- and postintervention assessments. This increase was evident in the total program (t_216_=2.59; *P*=.01; mean difference=0.45; 95% CI 0.12-0.80) and was particularly notable in the Jumpy program (t_55_=2.42; *P*=.02; mean difference=0.84; 95% CI 0.14-1.55) relative to the waitlist group (see Table S2 in Multimedia Appendix 1 for more details).

### Intervention Effectiveness

The effectiveness of the total, Pleaser, Shelly, and Jumpy programs was tested using repeated-measures ANOVA. As for the Riggy program, where the ages of the intervention and waitlist groups were significantly different, repeated-measures ANCOVA with age as a covariate was used to test its effectiveness.

#### Total Program

**Figure 2:**
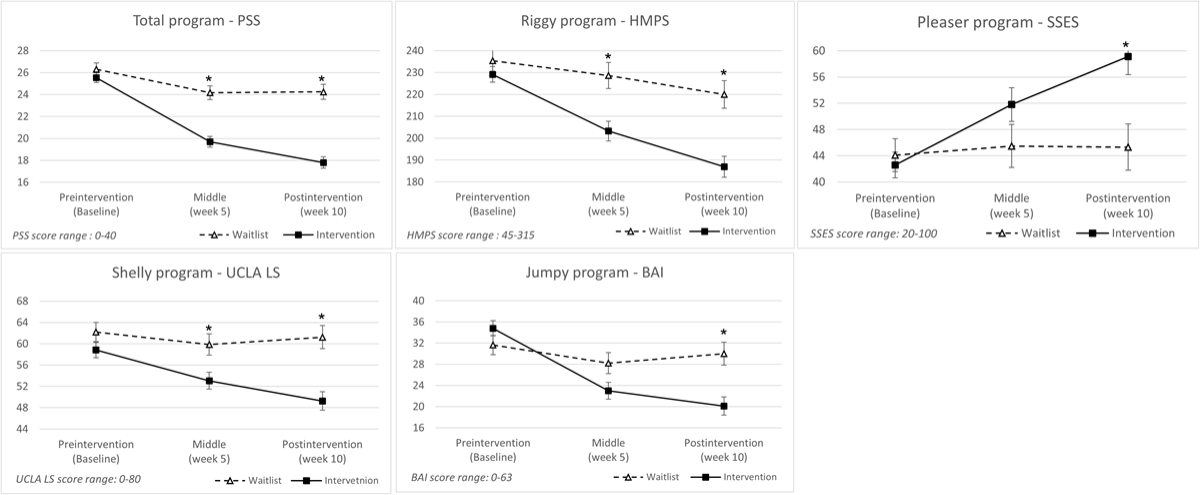
Primary outcomes at each assessment point. BAI: Beck Anxiety Inventory; HMPS: Hewitt Multidimensional Perfectionism Scale; PSS: Perceived Stress Scale; SSES: State Self-Esteem Scale; ULCA LS: University of California, Los Angeles Loneliness Scale.

In the total program, a significant time-by-group interaction was found for perceived stress (*F*_2,432_=27.52; *P*<.001; partial η^2^=0.11). There was also a significant main effect of time (*F*_2,432_=89.49; *P*<.001; partial η^2^=0.29) and a significant main effect of group (*F*_1,216_=35.19; *P*<.001; partial η^2^=0.14). Post hoc univariate ANOVAs indicated that the intervention group had significantly lower perceived stress scores than the waitlist group at the midintervention assessment (*F*_1,216_=32.01; *P*<.001; partial η^2^=0.13) and at the postintervention assessment (*F*_1,216_=55.32; *P*<.001; partial η^2^=0.20; Table 3; Figure 2).

Among the secondary outcomes, significant time-by-group interactions were observed for self-efficacy, depressive symptoms, and trait anxiety. Post hoc univariate ANCOVAs indicated that the intervention group scored significantly lower than the waitlist group on all 3 outcomes at both mid- and postintervention (Table S3 in Multimedia Appendix 1).

These results remained consistent when adjusting for mental health service use or psychiatric medication use via repeated-measures ANCOVA (Table S4 in Multimedia Appendix 1). The observed intervention effects were not attributable to differential service use across groups, as no significant interactions with mental health service use were found.

**Table 3:** Between-group comparisons for primary outcomes in each program.

Primary outcome (program), time point, and group				Participants, mean (SD)		*F* test (*df*)^a^		*P* value^b^		Partial η^2^
**PSS^c^ (total)**										
	**Baseline**				1.14 (1, 216)		.29		0.01	
		Intervention	25.53 (4.90)							
		Waitlist	26.3 (5.54)							
	**Week 5**				32.01 (1, 216)		<.001		0.13	
		Intervention	19.69 (5.33)							
		Waitlist	24.18 (6.16)							
	**Week 10**				55.32 (1, 216)		<.001		0.20	
		Intervention	17.80 (6.01)							
		Waitlist	24.25 (6.46)							
**HMPS^d^ (Riggy)**										
	**Baseline**				1.01 (1, 51)		.32		0.02	
		Intervention	230.21 (22.58)							
		Waitlist	233.55 (17.04)							
	**Week 5**				10.99 (1, 51)		.002		0.18	
		Intervention	204.82 (30.14)							
		Waitlist	225.9 (16.22)							
	**Week 10**				16.50 (1, 51)		<.001		0.24	
		Intervention	186.06 (31.47)							
		Waitlist	221.4 (17.07)							
**SSES^e^ (Pleaser)**										
	**Baseline**				0.23 (1, 48)		.63		<.01	
		Intervention	42.58 (9.59)							
		Waitlist	44.11 (12.81)							
	**Week 5**				2.32 (1, 48)		.13		0.05	
		Intervention	51.81 (13.63)							
		Waitlist	45.47 (15.29)							
	**Week 10**				9.49 (1, 48)		.003		0.17	
		Intervention	59.13 (13.99)							
		Waitlist	45.32 (17.49)							
**UCLA-LS^f^ (Shelly)**										
	**Baseline**				2.11 (1, 55)		.15		0.04	
		Intervention	55.83 (9.00)							
		Waitlist	62.18 (7.58)							
	**Week 5**				7.13 (1, 55)		.01		0.11	
		Intervention	53.06 (9.03)							
		Waitlist	59.86 (9.89)							
	**Week 10**				18.82 (1, 55)		<.001		0.25	
		Intervention	49.26 (9.82)							
		Waitlist	61.23 (10.65)							
**BAI^g^ (Jumpy)**										
	**Baseline**				0.87 (1, 55)		.36		0.02	
		Intervention	34.79 (12.39)							
		Waitlist	31.63 (11.34)							
	**Week 5**				2.30 (1, 55)		.14		0.04	
		Intervention	23 (12.13)							
		Waitlist	28.21 (12.39)							
	**Week 10**				6.76 (1, 55)		.01		0.11	
		Intervention	20.11 (13.38)							
		Waitlist	30 (13.90)							

^a^*F* test ratios were derived from univariate ANOVA for all programs, except for the Riggy program, for which univariate analysis of covariance, including age as a covariate was conducted.

^b^*P* values were Bonferroni adjusted and considered statistically significant at the .05 level (2-tailed).

^c^PSS: Perceived Stress Scale.

^d^HMPS: Hewitt Multidimensional Perfectionism Scale.

^e^SSES: State Self-Esteem Scale.

^f^UCLA-LS: University of California, Los Angeles Loneliness Scale.

^g^BAI: Beck Anxiety Inventory.

#### Riggy Program

In the Riggy program, repeated-measures ANCOVA with age as a covariate revealed a significant time-by-group interaction for perfectionism (*F*_2,102_=6.24; *P*=.003; partial η^2^=0.10). No significant main effect of time was observed (*F*_2,102_=1.70; *P*=.19; partial η^2^=0.03), whereas the main effect of group was significant (*F*_1,51_=8.95; *P*=.004; partial η^2^=0.15). A significant time-by-age interaction was also observed (*F*_2,102_=3.25; *P*=.04; η^2^=0.06), indicating that age may have moderated the intervention effect. Post hoc univariate ANCOVAs showed that the intervention group had significantly lower perfectionism scores than the waitlist group at midintervention (*F*_1,51_=10.99; *P*=.002; partial η^2^=0.18) and postintervention (*F*_1,51_=16.50; *P*<.001; partial η^2^=0.24; Table 3; Figure 2).

Among the secondary outcomes, significant time-by-group interactions were observed for perceived stress, self-efficacy, and trait anxiety, but not for depressive symptoms. Post hoc univariate ANCOVAs indicated that the intervention group scored significantly lower than the waitlist group on trait anxiety at both midintervention and postintervention. For perceived stress, the group difference was significant only at postintervention. Self-efficacy scores did not differ significantly between groups at either time point (Table S3 in Multimedia Appendix 1).

#### Pleaser Program

For the Pleaser program, repeated-measures ANOVA revealed a significant time-by-group interaction for state self-esteem (*F*_2,96_=11.83; *P*<.001; partial η^2^=0.20). There was also a significant main effect of time (*F*_2,96_=16.05; *P*<.001; partial η^2^=0.25) and a nonsignificant main effect of group (*F*_1,48_=3.08; *P*=.10; partial η^2^=0.06). Post hoc univariate ANOVAs indicated that the intervention group had significantly higher state self-esteem scores than the waitlist group at the postintervention assessment (*F*_1,48_=9.49; *P*=.003; partial η^2^=0.17; Table 3; Figure 2).

Among the secondary outcomes, significant time-by-group interactions were observed for perceived stress, depressive symptoms, and self-efficacy, but not for trait anxiety. Post hoc univariate ANOVAs indicated that the intervention group scored significantly lower than the waitlist group on perceived stress and depressive symptoms at both mid- and postintervention, and significantly higher on self-efficacy at postintervention (Table S6 in Multimedia Appendix 1).

#### Shelly Program

For the Shelly program, repeated-measures ANOVA revealed a significant time-by-group interaction for loneliness (*F*_2,110_=8.26; *P*<.001; partial η^2^=0.13). There was also a significant main effect of time (*F*_2,110_=13.34; *P*<.001; partial η^2^=0.20). The main effect of group was significant (*F*_1,55_=12.15; *P*<.001; partial η^2^=0.18). Post hoc univariate ANOVAs indicated that the intervention group reported significantly lower loneliness scores than the waitlist group at both the midintervention (*F*_1,55_=7.13; *P*=.01; partial η^2^=0.11) and postintervention assessments (*F*_1,55_=18.82; *P*<.001; partial η^2^=0.25; Table 3; Figure 2).

Among the secondary outcomes, significant time-by-group interactions were observed for perceived stress, depressive symptoms, trait anxiety, and self-efficacy. Post hoc univariate ANOVAs indicated that the intervention group scored significantly lower than the waitlist group on perceived stress, depressive symptoms, and trait anxiety, and significantly higher on self-efficacy at both mid- and postintervention assessments (Table S7 in Multimedia Appendix 1).

#### Jumpy Program

For the Jumpy program, repeated-measures ANOVA revealed a significant time-by-group interaction for anxiety (*F*_2,110_=10.75; *P*<.001; partial η^2^=0.16). There was also a significant main effect of time (*F*_2,110_=20.44; *P*<.001; partial η^2^=0.27). The main effect of group was nonsignificant (*F*_1,55_=1.61; *P*=.21; partial η^2^=0.03). Post hoc univariate ANOVAs indicated that the intervention group reported significantly lower anxiety scores than the waitlist group at the postintervention assessment (*F*_1,55_=6.76; *P*=.01; partial η^2^=0.11; Table 3; Figure 2).

Among the secondary outcomes, significant time-by-group interactions were observed for perceived stress, depressive symptoms, trait anxiety, and self-efficacy. Post hoc univariate ANOVAs indicated that the intervention group scored significantly lower than the waitlist group on perceived stress at both mid- and postintervention, and on depressive symptoms and trait anxiety at postintervention. Self-efficacy scores were significantly higher in the intervention group at postintervention (Table S8 in Multimedia Appendix 1).

These results remained consistent when adjusting for mental health service use via repeated-measures ANCOVA. Notably, the difference in trait anxiety between groups became significant at both mid- and postintervention after covariate adjustment, while the perceived stress findings remained stable (Table S9 in Multimedia Appendix 1). The observed intervention effects were not attributable to differential service use across groups, as no significant interactions with mental health service use were found.

### Maintenance of Intervention Effects

To check if the effects of the program lasted beyond its completion, the intervention group completed a postintervention assessment immediately after the program and a follow-up assessment 4 weeks later; a paired *t* test was conducted on the postintervention and follow-up assessment data.

No statistically significant mean differences were found between postintervention and follow-up scores for any of the primary outcomes, including perceived stress in the total program (t_87_=−0.68; *P*=.50), perfectionism in the Riggy program (t_20_=0.16; *P*=.88), self-esteem in the Pleaser program (t_16_=0.47; *P*=.64), loneliness in the Shelly program (t_23_=−0.18; *P*=.86), or anxiety in the Jumpy program (t_25_=−0.25; *P*=.81). Similarly, no significant differences between postintervention and follow-up were observed for most secondary outcomes. An exception was found in the Jumpy program, where participants in the intervention group showed a significant reduction in perceived stress from postintervention to follow-up, with a mean difference of 1.35 (95% CI 0.18-2.52; t_25_=2.37; *P*=.03; Table S10 in Multimedia Appendix 1). These findings suggest that the intervention effects were generally maintained over the 4-week follow-up period.

## Discussion

### Principal Findings

This study evaluated the effectiveness of a 10-week digital intervention program designed to reduce psychological distress and promote adaptive coping among adults experiencing recurring cognitive-emotional adaptation difficulties, such as perfectionism, low self-esteem, social isolation, and anxiety, that interfere with daily functioning. Rather than conceptualizing these issues as clinical diagnoses or stable personality traits, the intervention approached them as modifiable cognitive-emotional patterns shaped by early maladaptive schemas and maintained through habitual coping styles [14,15]. Participants were assigned to 1 of 4 intervention modules that corresponded to their dominant schema–driven pattern: Riggy for perfectionism, Pleaser for low self-esteem and fear of rejection, Shelly for social withdrawal, and Jumpy for heightened threat sensitivity. This schema-informed partial personalization approach aimed to match participants with content that aligned with their most salient cognitive-emotional vulnerabilities, offering a more psychologically resonant alternative to generic or diagnosis-focused interventions.

The primary aim of the study was to assess whether targeting these underlying schema-based patterns through structured digital intervention could lead to meaningful improvements in psychological well-being. Participants assigned to an active intervention condition showed greater improvements than those in a waitlist control group who had been matched to a schema-informed program but did not receive the intervention. Across all 4 modules, participants reported marked reductions in their dominant maladaptive patterns. Secondary outcomes also improved across domains such as perceived stress, depressive mood, trait anxiety, and self-efficacy. These gains were sustained at a 1-month follow-up, suggesting preliminary durability of the observed effects.

These findings can be interpreted in light of the intervention’s use of pattern-specific therapeutic techniques grounded in well-established psychological theories [9,10,16]. While many digital mental health programs focus primarily on symptom alleviation, the present intervention emphasized the root-level schemas and self-beliefs that generate and maintain distress. By delivering psychoeducation and behavioral strategies matched to users’ dominant distress patterns, the program likely enhanced emotional salience and internal relevance, factors known to drive meaningful engagement and change in digital formats [8,30]. For example, cognitive restructuring techniques targeting perfectionistic beliefs were central to the Riggy program, while exposure and value-driven action strategies were emphasized in the Pleaser program to address social fears, aligning with previous research on schema-relevant intervention mechanisms. The Shelly program focused on reducing social withdrawal through graded exposure and self-image restructuring, whereas the Jumpy program incorporated anxiety regulation skills and exposure-based coping to address hypervigilance and threat sensitivity.

According to the predictive coding theory, these improvements may also reflect processes of corrective learning via schema-disconfirming experiences. By repeatedly exposing users to structured content that challenged deeply held but maladaptive expectations, such as the inevitability of failure or rejection, the program may have generated prediction errors that gradually weakened the influence of deep-seated cognitive-emotional patterns [14]. In this way, schema-informed personalization was not only about improving engagement but about targeting the underlying belief systems that perpetuate distress. This interpretation is further supported by recent ecological momentary assessment studies showing that the activation of such patterns can vary meaningfully across daily contexts [15], suggesting that these tendencies, once considered rigid, are more dynamic and environmentally sensitive than previously thought. In this light, the high accessibility and frequency of digital interventions may provide users with more opportunities to encounter schema-disconfirming input in real-time moments of vulnerability, facilitating meaningful psychological updating.

Although this intervention focused on cognitive-emotional patterns rather than psychiatric disorders, the observed effects suggest potential value for early mental health support. Schema-linked difficulties, such as perfectionism, rejection sensitivity, social withdrawal, and anxious overcontrol, are well-known contributors to long-term distress and functional impairment if left unaddressed [10]. Improvements in these domains, alongside increased self-efficacy, may reflect gains in coping capacity and psychological resilience in everyday life. Although long-term outcomes remain to be assessed, schema-informed digital programs such as this one may serve as accessible tools for addressing modifiable psychological patterns in individuals who may not seek or require formal clinical treatment [13]. In this context, digital interventions could play a meaningful role within stepped-care or prevention-oriented mental health systems [31].

In terms of adherence, among the 187 participants who logged into the platform and did not withdraw before baseline, 94.7% (177/187) engaged in at least 1 session and 70.1% (131/187) completed all 10 sessions. These figures are notably higher than completion rates typically observed in digital mental health interventions, where dropout rates commonly range from 23% to 64% and completion rates vary widely from 26% to 100% depending on intervention characteristics and population [29,32,33]**.** The 70.1% (131/187) completion rate achieved in this study places it in the upper quartile of digital mental health intervention’s adherence outcomes. While the primary outcome analyses were based on a more conservative per-protocol sample (218/300, 72.7%), these broader adherence data offer preliminary support for the feasibility and engagement potential of schema-based, pattern-aligned digital interventions in psychologically distressed adults.

The intervention’s schema-informed personalization likely increased emotional salience and perceived relevance by aligning content with users’ dominant psychological patterns, such as perfectionism or rejection sensitivity. These familiar themes may have helped users feel recognized and validated, thereby facilitating continued engagement. While this study design does not permit definitive causal attribution to the personalization component specifically, the observed patterns are consistent with conceptual frameworks, suggesting that content alignment with users’ cognitive-emotional profiles enhances engagement [8,34].

Beyond content personalization, the high engagement rates observed may also be partially explained by elements consistent with the supportive-accountability framework [35], where structured content delivery, progress tracking, and clear expectations create a sense of commitment even in the absence of direct human support. In addition, by fostering a perceived fit between users’ schema-linked distress patterns and the intervention material, the program may have supported a digitally mediated form of therapeutic alliance, often considered a key predictor of outcome in traditional therapy contexts.

In addition, 941 individuals completed the initial online screening, over 3 times the intended recruitment target, indicating substantial demand for low-threshold, psychologically relevant digital support [32,34]. These reach and engagement figures underscore the practical advantages of digital platforms, which offer enhanced accessibility through on-demand access, reduced stigma, and minimal logistical barriers [31]. This observation aligns with findings from recent research on online mental health communities, where individuals frequently express distress rooted in dysfunctional core beliefs, many of which correspond to early maladaptive schemas [13]. Such platforms demonstrate both the demand for accessible emotional support and the prominence of schema-related cognitive-emotional patterns in naturally occurring digital discourse. Together, these findings suggest that schema-informed digital interventions can serve as both scalable and acceptable intervention formats in general population mental health efforts.

It is also consistent with the stepped-care model framework, where digital interventions serve as lower-intensity entry points to more comprehensive treatment systems [31]. Schema-aligned programs are particularly well suited to this role, as they target foundational psychological vulnerabilities that often precede diagnosable disorders. While these interventions effectively address early-stage distress, they naturally complement rather than replace traditional therapy for more complex cases. The absence of real-time therapist involvement reduces opportunities for relational attunement, contextual sensitivity, and clinical adaptability, elements that are central to effective in-person care [36]. In this sense, schema-informed digital interventions can be understood as psychologically attuned entry-level tools that reduce early distress, support coping, and help users identify when higher-intensity care is needed.

### Limitations and Future Research

This study has several limitations that should be acknowledged. First, it was conducted entirely on the web without face-to-face clinical interviews, which may have limited the precision with which participants’ symptom severity was assessed. In addition, the sample was predominantly female and highly educated, which may limit the generalizability of the findings to more diverse populations.

Second, despite the randomization of intervention and waitlist groups in each program, significant between-group differences in age and some secondary outcome measures were observed at baseline. For example, in the Riggy program, the intervention group was significantly older than the waitlist group and age could affect familiarity with and access to an online intervention program. To address this, repeated-measures ANCOVAs were conducted, indicating that while age had some influence on baseline perfectionist tendencies, the group×time effect remained significant across most outcomes, supporting the intervention’s effectiveness even after statistical adjustment.

Third, the use of additional mental health services or psychiatric medications during the intervention period was neither restricted nor systematically monitored. While ethical considerations preclude limiting access to care, such factors may have influenced outcome measures. In certain tracks (eg, Jumpy), intervention participants were more likely to access external services; however, post hoc analyses adjusting for service use indicated that the intervention effects remained robust. Future studies should consider incorporating systematic tracking of service use and examining its moderating or mediating role in outcome change.

Fourth, the study evaluated outcomes only up to 1-month postintervention. Given the intervention’s focus on relatively stable cognitive-emotional patterns, such as perfectionism and social isolation, it remains uncertain whether observed gains persist over longer durations. Future research should assess long-term outcomes at 6- or 12-month follow-up points to better evaluate durability and clinical significance.

Finally, while this study used a schema-informed personalization approach, assigning participants to programs based on their dominant cognitive-emotional pattern, it lacked a comparison condition involving nonpersonalized or mismatched content. As such, the specific contribution of the personalization component could not be isolated. Although the waitlist group was schema-matched at baseline, they did not receive active intervention. To more rigorously assess the impact of tailoring, future trials should directly compare personalized, nonpersonalized, and mismatched program versions. In addition, incorporating process-level metrics (eg, perceived fit, emotional resonance, and user satisfaction) would help clarify the mechanisms through which personalization influences engagement and outcomes [8].

### Conclusions

Despite its limitations, this study offers important contributions to the evolving landscape of digital mental health care. Rather than targeting clinical disorders or isolated symptoms, the intervention focused on schema-linked cognitive-emotional patterns that commonly affect the general population. Using a schema-based partial personalization framework, the program aligned intervention content with participants’ dominant patterns, aiming to reduce distress and support adaptive coping in a scalable and accessible digital format. While definitive conclusions about the causal role of personalization cannot yet be drawn, the pattern of findings suggests meaningful psychological resonance and potential utility. This points to the value of such tools as early-stage supports within stepped-care systems, particularly for individuals not engaged in formal treatment. By extending mental health support to nonclinical populations and embedding personalization into digital delivery, this study contributes to building more inclusive, adaptive, and process-sensitive approaches to psychological care.

## Appendix

Multimedia Appendix 1 Supplementary statistical results referenced in the Results section.

Multimedia Appendix 2 CONSORT-eHEALTH checklist (V 1.6.1).

## Abbreviations

ACT: acceptance and commitment therapy
ANCOVA: analyses of covariance
BAI: Beck Anxiety Inventory
CBT: cognitive behavioral therapy
HMPS: Hewitt Multidimensional Perfectionism Scale
IRB: institutional review board
MBCT: mindfulness-based cognitive therapy
PSS-10: 10-item Perceived Stress Scale
SSES: State Self-Esteem Scale

## Data Availability

The datasets used and analyzed during this study are available from the corresponding author on reasonable request.
