# Correction: Schema-Informed Digital Mental Health Intervention for Maladaptive Cognitive-Emotional Patterns: Randomized Controlled Trial

**DOI:** 10.2196/83135

**Published:** 2025-09-17

**Authors:** Seohyun Jeong, Hyeonseong Kim, Silvia Kyungjin Lho, Inae Hwang, Seongjun Mun, Soohyun Kim, Hyejin Lim, Hyeonhee Kim, Min-Sup Shin, Woori Moon

**Affiliations:** 1 Department of Psychiatry Seoul National University Hospital Seoul Republic of Korea; 2 40FY Inc Seoul Republic of Korea; 3 Department of Psychiatry Seoul Metropolitan Government-Seoul National University Boramae Medical Center Seoul Republic of Korea; 4 Seoul National University College of Medicine Seoul Republic of Korea

In “Schema-Informed Digital Mental Health Intervention for Maladaptive Cognitive-Emotional Patterns: Randomized Controlled Trial” (J Med Internet Res 2025;27:e65892) the authors made a few corrections to Table 1:

In the “Total program” row, the Age (years) value was:

1.95 (8.09; 18–58)

It has been revised to:

31.95 (8.09; 18–58)

In addition, in Table 1, the following corrections were made:

- Removed the word “program” so the row reads “Total” only.
- Removed percentage values for the “Total” row to match other rows.
- Reordered the rows as Total – Riggy – Pleaser – Shelly – Jumpy.

The correction will appear in the online version of the paper on the JMIR Publications website together with the publication of this correction notice. Because this was made after submission to PubMed, PubMed Central, and other full-text repositories, the corrected article has also been resubmitted to those repositories.

