# Supplementary material for: Schema-Informed Digital Mental Health Intervention for Maladaptive Cognitive-Emotional Patterns: Randomized Controlled Trial
Source: J Med Internet Res. 2025 Aug 14;27:e65892. doi: 10.2196/65892 (PMC12395104; doi:10.2196/65892)
Supplement: Multimedia Appendix 1 [file jmir_v27i1e65892_app1.docx]

**Index**

Supplemental Table 1. Primary and secondary outcomes at pre-intervention (baseline) p. 2

Supplemental Table 2. Mental health service use and prescription medications during the intervention period p. 3

Supplemental Table 3. Impact of personality trait focused digital intervention – Total program (secondary outcome results)

p. 4

Supplemental Table 4. Impact of personality trait focused digital intervention adjusting for mental health service use during intervention period – Total program p. 5

Supplemental Table 5. Impact of personality trait focused digital intervention – Riggy program (secondary outcome results)

p. 6

Supplemental Table 6. Impact of personality trait focused digital intervention – Pleaser program (secondary outcome results)

p. 7

Supplemental Table 7. Impact of personality trait focused digital intervention – Shelly program (secondary outcome results)

p. 8

Supplemental Table 8. Impact of personality trait focused digital intervention – Jumpy program (secondary outcome results)

p. 9

Supplemental Table 9. Impact of personality trait focused digital intervention adjusting for mental health service use during intervention period – Jumpy program p. 10

Supplemental Table 10. Maintenance of intervention effects p. 11

Supplemental Figure 1. Secondary results at each assessment point – Total program p. 12

Supplemental Figure 2. Secondary results at each assessment point – Riggy program p. 13

Supplemental Figure 3. Secondary results at each assessment point – Pleaser program p. 14

Supplemental Figure 4. Secondary results at each assessment point – Shelly program p. 15

Supplemental Figure 5. Secondary results at each assessment point – Jumpy program p. 16

**Supplemental Table 1. Primary and secondary outcomes at pre-intervention (baseline)**

| Total Program | | | | | |  |
| --- | --- | --- | --- | --- | --- | --- |
|  | Total  *N* = 218 | Intervention  *N* = 138 | Waitlist  *N* = 80 | *t* | *P* |  |
| *Primary outcome measure* | | | | | |  |
| PSS | 25.81±5.15 | 25.53±4.90 | 26.30±5.55 | -1.07 | .29 |  |
| *Secondary outcome measure* | | | | | |  |
| SES | 63.44±15.63 | 63.36±15.42 | 63.58±16.07 | -.10 | .92 |  |
| CES-D | 30.54±11.9 | 30.18±11.53 | 31.15±12.56 | -.58 | .56 |  |
| STAI trait | 58.33±10.37 | 58.15±10.06 | 58.63±10.92 | -.32 | .75 |  |
| Riggy Program | | | | | |  |
|  | Total  *N* = 54 | Intervention  *N* = 34 | Waitlist  *N* = 20 | *t* | *P* |  |
| *Primary outcome measure* | | | | | |  |
| HMPS | 231.44±20.60 | 230.21±22.58 | 233.55±17.04 | -.57 | .57 |  |
| *Secondary outcome measure* | | | | | |  |
| PSS | 24.76±5.56 | 24.47±5.23 | 25.25±6.18 | 1.27 | .21 |  |
| SES | 74.81±16.23 | 74.06±17.04 | 76.10±15.08 | -.44 | .66 |  |
| CES-D | 26.15±12.02 | 25.53±11.13 | 27.20±13.64 | -.49 | .63 |  |
| STAI trait | 54.52±11.34 | 53.79±11.17 | 55.75±11.81 | -.61 | .55 |  |
| Pleaser Program | | | | | |  |
|  | Total  *N* = 50 | Intervention  *N* = 31 | Waitlist  *N* = 19 | *t* | *P* |  |
| *Primary outcome measure* | | | | | |  |
| SSES | 43.16±10.83 | 42.58±9.59 | 44.11±12.81 | -.45 | .63 |  |
| *Secondary outcome measure* | | | | | |  |
| PSS | 26±4.24 | 25.97±3.95 | 26.05±4.80 | -.07 | .95 |  |
| SES | 57.52±12.57 | 56.39±11.14 | 59.37±14.74 | -.81 | .95 |  |
| CES-D | 29.7±10.81 | 29.06±10.52 | 30.74±11.47 | -.53 | .60 |  |
| STAI trait | 59.5±9.65 | 60.87±8.34 | 57.26±11.36 | 1.29 | .20 |  |
| Shelly Program | | | | | |  |
|  | Total  *N* = 57 | Intervention  *N* = 35 | Waitlist  *N* = 22 | *t* | *P* |  |
| *Primary outcome measure* | | | | | |  |
| UCLA LS | 60.12±8.57 | 58.83±9.00 | 62.18±7.58 | -1.45 | .15 |  |
| *Secondary outcome measure* | | | | | |  |
| PSS | 25.63±5.21 | 25.06±4.99 | 26.55±5.53 | -1.05 | .30 |  |
| SES | 59.35±12.19 | 60.97±12.80 | 56.77±10.95 | 1.27 | .21 |  |
| CES-D | 31.11±10.51 | 30.77±9.71 | 31.64±11.90 | -.30 | .77 |  |
| STAI trait | 58.37±9.28 | 57.40±9.22 | 59.91±9.39 | -.99 | .33 |  |
| Jumpy Program | | | | | |  |
|  | Total  *N* = 57 | Intervention  *N* = 38 | Waitlist  *N* = 19 | *t* | *P* |  |
| *Primary outcome measure* | | | | | |  |
| BAI | 33.74±12.05 | 34.79±12.40 | 31.63±11.34 | .93 | .36 |  |
| *Secondary outcome measure* | | | | | |  |
| PSS | 26.82±5.33 | 26.55±5.15 | 27.37±5.78 | -.54 | .59 |  |
| SES | 61.93±15.33 | 61.66±14.71 | 62.47±16.90 | -.19 | .85 |  |
| CES-D | 34.86±12.68 | 34.71±12.78 | 35,16±12.84 | -.12 | .90 |  |
| STAI trait | 60.86±10.26 | 60.53±9.97 | 61.53±11.07 | -.34 | .73 |  |

Note. PSS = Perceived Stress Scale. SES = Self-Efficacy Scale. CES-D = Center for Epidemiologic Studies Depression Scale. STAI trait = Trait anxiety of State-trait Anxiety Inventory. SSES = State Self-Esteem Scale. UCLA-LS = UCLA Loneliness Scale. BAI = Beck Anxiety Index.

**Supplemental Table 2. Mental health service use and prescription medications during the intervention period**

| Program |  | Total  *M*±*SD* | Intervention  *M*±*SD* | Waitlist  *M*±*SD* | *t* | *P* |
| --- | --- | --- | --- | --- | --- | --- |
| Total  *N* = 218 | Mental health services | .94±1.30 | 1.10±1.34 | .65±1.18 | 2.59 | .01 |
|  | Prescription medications | .69±1.20 | .80±1.25 | .50±1.09 | 1.88 | .06 |
| Riggy  *N* = 54 | Mental health services | .67±1.15 | .79±1.23 | .65±1.20 | 1.06 | .29 |
|  | Prescription medications | .52±1.08 | .65±1.20 | .30±.80 | 1.27 | .21 |
| Pleaser  *N* = 50 | Mental health services | .84±1.25 | 1.06±1.34 | .47±1.02 | 1.76 | .09 |
|  | Prescription medications | .62±1.16 | .77±1.26 | .37±.96 | 1.29 | .20 |
| Shelly  *N* = 57 | Mental health services | 1.07±1.39 | 1.09±1.38 | 1.05±1.43 | .11 | .92 |
|  | Prescription medications | .75±1.26 | .71±1.20 | .82±1.37 | -.30 | .76 |
| Jumpy  *N* = 57 | Mental health services | 1.14±1.36 | 1.42±1.37 | .58±1.17 | 2.42 | .02 |
|  | Prescription medications | .86±1.30 | 1.05±1.35 | .47±1.12 | 1.71 | .10 |

Note. The level of mental health service use and prescription medication during the intervention period were computed as the sum of the number of times respondents reported using mental health services and prescription medication in the assessments at weeks 0, 5, and 10.

**Supplemental Table 3. Impact of personality trait focused digital intervention – Total program (secondary outcome results)**

|  |  | Statistics  *F*; *P-*value | Pre-intervention  (baseline)  *M* ± *SD* | Middle  (Week 5)  *M* ± *SD* | Post-intervention  (Week 10)  *M* ± *SD* |
| --- | --- | --- | --- | --- | --- |
| SES | Time | 17.81; < .001 |  |  |  |
|  | Group | 8.60; .004 | *F* = .01; *P* = .92 | *F* = 8.7*;* *P* = .004 | *F* = 26.79; *P* < .001 |
|  | Intervention |  | 63.36 ± 15.42 | 69.51 ± 14.49 | 73.72 ± 15.04 |
|  | Waitlist |  | 63.58 ± 16.07 | 63.3 ± 15.83 | 62.16 ± 17.26 |
|  | Group*Time | 29.52; < .001 |  |  |  |
| CES-D | Time | 37.86; < .001 |  |  |  |
|  | Group | 15.90; < .001 | *F* = .33; *P* = .56 | *F* = 13.44; *P* < .001 | *F* = 36.65; *P* < .001 |
|  | Intervention |  | 30.18 ± 11.53 | 22.68 ± 11.43 | 19.3 ± 12.29 |
|  | Waitlist |  | 31.15 ± 12.56 | 29.01 ± 13.66 | 30.28 ± 13.89 |
|  | Group*Time | 24.17; < .001 |  |  |  |
| STAI trait | Time | 34.07; < .001 |  |  |  |
|  | Group | 15.90; < .001 | *F* = .10; *P* = .75 | *F* = 17.81; *P* < .001 | *F* = 32.37; *P* < .001 |
|  | Intervention |  | 58.15 ± 10.06 | 51.75 ± 10.72 | 48.49 ± 11.66 |
|  | Waitlist |  | 58.63 ± 10.92 | 58.33 ± 11.68 | 57.99 ± 12.26 |
|  | Group*Time | 26.44; < .001 |  |  |  |

Note: In the *Statistics* column, *F*-ratios were derived from repeated measures analysis of variance (ANOVA) models. In the *Pre-intervention*, *Middle*, and *Post-intervention* columns, F-ratios were derived from univariate ANOVA models. *M* = mean. *SD* = standard deviation. PSS = Perceived Stress Scale. CES-D = Center for Epidemiologic Studies Depression Scale. SES = Self-Efficacy Scale. STAI trait = Trait anxiety of State-trait Anxiety Inventory.

**Supplemental Table 4. Impact of personality trait focused digital intervention adjusting for mental health service use during intervention period – Total program**

|  |  | Statistics  *F;* *P-*value | Pre-intervention  (baseline)  *M* ± *SD* | Middle  (Week 5)  *M* ± *SD* | Post-intervention  (Week 10)  *M* ± *SD* |
| --- | --- | --- | --- | --- | --- |
| PSS | Time | 60.67; < .001 |  |  |  |
|  | Mental health service | .97; .33 |  |  |  |
|  | Group | 36.15; < .001 | *F* = 1.53; *P* = .22 | *F* = 31.92; *P* < .001 | *F* = 55.90; *P* < .001 |
|  | Intervention |  | 25.53 ± 4.9 | 19.69 ± 5.33 | 17.8 ± 6.01 |
|  | Waitlist |  | 26.3 ± 5.54 | 24.18 ± 6.16 | 24.25 ± 6.46 |
|  | Mental health service *Time | .17; .84 |  |  |  |
|  | Group*Time | 26.49; < .001 |  |  |  |
| SES | Time | 9.59; < .001 |  |  |  |
|  | Mental health service | 3.95; .04 |  |  |  |
|  | Group | 10.52; .001 | *F* = .08; *P* = .78 | *F* = 10.33; *P* = .002 | *F* = 28.78; *P* < .001 |
|  | Intervention |  | 63.36 ± 15.42 | 69.51 ± 14.49 | 73.72 ± 15.04 |
|  | Waitlist |  | 63.58 ± 16.07 | 63.3 ± 15.83 | 62.16 ± 17.26 |
|  | Mental health service *Time | .59; .52 |  |  |  |
|  | Group*Time | 27.70; < .001 |  |  |  |
| CES-D | Time | 25.99; < .001 |  |  |  |
|  | Mental health service | 3.47; .06 |  |  |  |
|  | Group | 18.2; < .001 | *F* = .76; *P* = .38 | *F* = 14.8; *P* < .001 | *F* = 39.61; *P* < .001 |
|  | Intervention |  | 30.18 ± 11.53 | 22.68 ± 11.43 | 19.3 ± 12.29 |
|  | Waitlist |  | 31.15 ± 12.56 | 29.01 ± 13.66 | 30.28 ± 13.89 |
|  | Mental health service *Time | .19; .796 |  |  |  |
|  | Group*Time | 23.54; < .001 |  |  |  |
| STAI trait | Time | 17.2; < .001 |  |  |  |
|  | Mental health service | 8.4; .004 |  |  |  |
|  | Group | 20.11; < .001 | *F* = .95; *P* = .33 | *F* = 20.58; *P* < .001 | *F* = 35.64; *P* < .001 |
|  | Intervention |  | 58.15 ± 10.06 | 51.75 ± 10.72 | 48.49 ± 11.66 |
|  | Waitlist |  | 58.63 ± 10.92 | 58.33 ± 11.68 | 57.99 ± 12.26 |
|  | Mental health service *Time | 2.07; .13 |  |  |  |
|  | Group*Time | 23.56; < .001 |  |  |  |

Note: In the *Statistics* column, *F*-ratios were derived from repeated measures analysis of covariance (ANCOVA) models which included mental health service use during intervention period as a covariate. In the *Pre-intervention*, *Middle*, and *Post-intervention* columns, *F*-ratios were derived from univariate ANCOVA models. *M* = mean. *SD* = standard deviation. PSS = Perceived Stress Scale. CES-D = Center for Epidemiologic Studies Depression Scale. SES = Self-Efficacy Scale. STAI trait = Trait anxiety of State-trait Anxiety Inventory.

**Supplemental Table 5. Impact of personality trait focused digital intervention – Riggy program (secondary outcome results)**

|  |  | Statistics  *F;* *p-*value | Pre-intervention  (baseline)  *M* ± *SD* | Middle  (Week 5)  *M* ± *SD* | Post-intervention  (Week 10)  *M* ± *SD* |
| --- | --- | --- | --- | --- | --- |
| PSS | Time | .05; .95 |  |  |  |
|  | Age | .01; .92 |  |  |  |
|  | Group | 6.8; .01 | *F* = .52; *P* = .47 | *F* = 2.45; *P* = .12 | *F* = 13.29; *P* < .001 |
|  | Intervention |  | 24.47 ± 5.23 | 19.38 ± 5.67 | 16.47 ± 5.56 |
|  | Waitlist |  | 25.25 ± 6.18 | 22.4 ± 6.01 | 23.1 ± 6.05 |
|  | Age*Time | .81; .45 |  |  |  |
|  | Group*Time | 3.68; .03 |  |  |  |
| SES | Time | .70; .46 |  |  |  |
|  | Age | .08; .77 |  |  |  |
|  | Group | .77; .38 | *F* = .38; *P* = .54 | *F* = 2.06; *P* = .16 | *F* = 3.1; *P* = .08 |
|  | Intervention |  | 74.06 ± 17.04 | 79.03 ± 14.48 | 81.82 ± 13.46 |
|  | Waitlist |  | 76.1 ± 15.08 | 72.75 ± 14.78 | 74.25 ± 14.28 |
|  | Age*Time | .42; .60 |  |  |  |
|  | Group*Time | 5.22; .01 |  |  |  |
| CES-D | Time | .18; .79 |  |  |  |
|  | Mental health service | .23; .64 |  |  |  |
|  | Group | 5.12; .03 | *F* = .44; *P* = .51 | *F* = 4.42; *P* = .04 | *F* = 8.86; *P* = .004 |
|  | Intervention |  | 25.53 ± 11.13 | 18.47 ± 11.49 | 14.97 ± 10.93 |
|  | Waitlist |  | 27.2 ± 13.64 | 25.45 ± 12.8 | 25.05 ± 11.81 |
|  | Age*Time | .20; .78 |  |  |  |
|  | Group*Time | 2.68; .08 |  |  |  |
| STAI trait | Time | 2.27; .12 |  |  |  |
|  | Age | .61; .44 |  |  |  |
|  | Group | 3.36; .07 | *F* = .02; *P* = .88 | *F* = 4.32; *P* = .04 | *F* = 6.77; *P* = .01 |
|  | Intervention |  | 53.79 ± 11.17 | 47.74 ± 10.57 | 44.32 ± 11.14 |
|  | Waitlist |  | 55.75 ± 11.81 | 54.95 ± 11.97 | 53.7 ± 10.98 |
|  | Age *Time | .8; .43 |  |  |  |
|  | Group*Time | 4.64; .02 |  |  |  |

Note: In the *Statistics* column, *F*-ratios were derived from repeated measures analysis of covariance (ANCOVA) models which included age as a covariate. In the *Pre-intervention*, *Middle*, and *Post-intervention* columns, *F*-ratios were derived from univariate ANCOVA models. *M* = mean. *SD* = standard deviation. PSS = Perceived Stress Scale. CES-D = Center for Epidemiologic Studies Depression Scale. SES = Self-Efficacy Scale. STAI trait = Trait anxiety of State-trait Anxiety Inventory.

**Supplemental Table 6. Impact of personality trait focused digital intervention – Pleaser program (secondary outcome results)**

|  |  | Statistics  *F;* *p-*value | Pre-intervention  (baseline)  *M* ± *SD* | Middle  (Week 5)  *M* ± *SD* | Post-intervention  (Week 10)  *M* ± *SD* |
| --- | --- | --- | --- | --- | --- |
| PSS | Time | 20.65; < .001 |  |  |  |
|  | Group | 5.44; .02 | *F* < .01; *P* = .95 | *F* = 5.59; *P* = .02 | *F* = 8.99; *P* = .004 |
|  | Intervention |  | 25.97 ± 3.95 | 20.81 ± 4.83 | 18.29 ± 6.66 |
|  | Waitlist |  | 26.05 ± 4.8 | 24.58 ± 6.41 | 24.53 ± 7.86 |
|  | Group*Time | 8.78; .001 |  |  |  |
| SES | Time | 7.34; .002 |  |  |  |
|  | Group | 1.51; .23 | *F* = .66; *P* = .42 | *F* = 1.07; *P* = .31 | *F* = 7.53; *P* = .009 |
|  | Intervention |  | 56.39 ± 11.14 | 62.68 ± 11.44 | 69.81 ± 14.46 |
|  | Waitlist |  | 59.37 ± 14.74 | 58.74 ± 15.47 | 57.37 ± 17.23 |
|  | Group*Time | 13.42; < .001 |  |  |  |
| CES-D | Time | 7.38; .002 |  |  |  |
|  | Group | 4.93; .03 | *F* = .28; *P* = .60 | *F* = 4.2; *P* = .04 | *F* = 10.71; *P* = .002 |
|  | Intervention |  | 29.06 ± 10.52 | 23 ± 10.23 | 19 ± 11.12 |
|  | Waitlist |  | 30.74 ± 11.47 | 30.37 ± 15.23 | 31.42 ± 15.69 |
|  | Group*Time | 9.27; < .001 |  |  |  |
| STAI trait | Time | 6.71; .004 |  |  |  |
|  | Group | .46; .50 | *F* = 1.67; *P* = .20 | *F* = .64; *P* = .43 | *F* = 3.67; *P* = .06 |
|  | Intervention |  | 60.87 ± 8.34 | 55.74 ± 9.44 | 51.23 ± 10.92 |
|  | Waitlist |  | 57.26 ± 11.36 | 58.21 ± 12.24 | 58.21 ± 14.78 |
|  | Group*Time | 10.01; < .001 |  |  |  |

Note: In the *Statistics* column, *F*-ratios were derived from repeated measures analysis of variance (ANOVA) models. In the *Pre-intervention*, *Middle*, and *Post-intervention* columns, *F*-ratios were derived from univariate ANOVA models. *M* = mean. *SD* = standard deviation. PSS = Perceived Stress Scale. CES-D = Center for Epidemiologic Studies Depression Scale. SES = Self-Efficacy Scale. STAI trait = Trait anxiety of State-trait Anxiety Inventory.

**Supplemental Table 7. Impact of personality trait focused digital intervention – Shelly program (secondary outcome results)**

|  |  | Statistics  *F;* *p-*value | Pre-intervention  (baseline)  *M* ± *SD* | Middle  (Week 5)  *M* ± *SD* | Post-intervention  (Week 10)  *M* ± *SD* |
| --- | --- | --- | --- | --- | --- |
| PSS | Time | 25.57; < .001 |  |  |  |
|  | Group | 14.75; < .001 | *F* = 1.11; *P* = .23 | *F* = 13.44; *P* = .001 | *F* = 22.37; *P* < .001 |
|  | Intervention |  | 25.06 ± 4.99 | 18.97 ± 5.41 | 17.34 ± 5.41 |
|  | Waitlist |  | 26.55 ± 5.53 | 24.77 ± 6.42 | 24.55 ± 5.89 |
|  | Group*Time | 8.53; .001 |  |  |  |
| SES | Time | 4.28; .02 |  |  |  |
|  | Group | 9.43; .003 | *F* = 1.62; *P* = .21 | *F* = 7.27; *P* = .009 | *F* = 16.55; *P* < .001 |
|  | Intervention |  | 60.97 ± 12.8 | 66.8 ± 14.02 | 71.29 ± 16.32 |
|  | Waitlist |  | 56.77 ± 10.95 | 57.05 ± 12.03 | 54.05 ± 14.29 |
|  | Group*Time | 11.34; < .001 |  |  |  |
| CES-D | Time | 14.1; < .001 |  |  |  |
|  | Group | 7.44; .01 | *F* = .09; *P* = .77 | *F* = 6.27; *P* = .02 | *F* = 18.37; *P* < .001 |
|  | Intervention |  | 30.77 ± 9.7 | 22.34 ± 10.70 | 18.34 ± 9.94 |
|  | Waitlist |  | 31.64 ± 11.9 | 30.32 ± 13.17 | 31.36 ± 12.91 |
|  | Group*Time | 11.9; < .001 |  |  |  |
| STAI trait | Time | 9.54; < .001 |  |  |  |
|  | Group | 11.22; .002 | *F* = .99; *P* = .33 | *F* = 14.05; *P* < .001 | *F* = 13.03; *P* < .001 |
|  | Intervention |  | 57.4 ± 9.22 | 50.14 ± 9.90 | 47.63 ± 10.90 |
|  | Waitlist |  | 59.91 ± 9.39 | 60.68 ± 11 | 58.68 ± 11.81 |
|  | Group*Time | 7.17; .002 |  |  |  |

Note: In the *Statistics* column, *F*-ratios were derived from repeated measures analysis of variance (ANOVA) models. In the *Pre-intervention*, *Middle*, and *Post-intervention* columns, *F*-ratios were derived from univariate ANOVA models. *M* = mean. *SD* = standard deviation. PSS = Perceived Stress Scale. CES-D = Center for Epidemiologic Studies Depression Scale. SES = Self-Efficacy Scale. STAI trait = Trait anxiety of State-trait Anxiety Inventory.

**Supplemental Table 8. Impact of personality trait focused digital intervention – Jumpy program (secondary outcome results)**

|  |  | Statistics  *F;* *p-*value | Pre-intervention  (baseline)  *M* ± *SD* | Middle  (Week 5)  *M* ± *SD* | Post-intervention  (Week 10)  *M* ± *SD* |
| --- | --- | --- | --- | --- | --- |
| PSS | Time | 27.11; < .001 |  |  |  |
|  | Group | 8.56; .005 | *F* = .29; *P* = .59 | *F* = 11.34; *P* = .001 | *F* = 10.89; *P* = .002 |
|  | Intervention |  | 26.55 ± 5.15 | 19.71 ± 5.37 | 19 ± 6.29 |
|  | Waitlist |  | 27.37 ± 5.78 | 24.95 ± 5.85 | 24.84 ± 6.32 |
|  | Group*Time | 6.51; .003 |  |  |  |
| SES | Time | 7.78; .002 |  |  |  |
|  | Group | 1.01; .32 | *F* = .04; *P* = .85 | *F* = .92; *P* = .34 | *F* = 4.03; *P* < .05 |
|  | Intervention |  | 61.66 ± 14.71 | 69.08 ± 13.19 | 71.89 ± 13.39 |
|  | Waitlist |  | 62.47 ± 16.90 | 65.16 ± 16.98 | 63.63 ± 16.95 |
|  | Group*Time | 4.12; .03 |  |  |  |
| CES-D | Time | 10.10; < .001 |  |  |  |
|  | Group | 1.75; .19 | *F* = .02; *P* = .90 | *F* = .91; *P* = .34 | *F* = 4.79; *P* = .033 |
|  | Intervention |  | 34.71 ± 12.78 | 26.5 ± 12.02 | 24.32 ± 14.74 |
|  | Waitlist |  | 35.16 ± 12.84 | 29.89 ± 13.87 | 33.37 ± 14.69 |
|  | Group*Time | 3.49; .04 |  |  |  |
| STAI trait | Time | 8.84; < .001 |  |  |  |
|  | Group | 4.30; .04 | *F* = .12; *P* = .73 | *F* = 3.11; *P* = .08 | *F* = 10; *P* = .003 |
|  | Intervention |  | 60.53 ± 9.97 | 53.58 ± 11.38 | 50.76 ± 12.61 |
|  | Waitlist |  | 61.53 ± 11.07 | 59.26 ± 11.69 | 61.47 ± 10.82 |
|  | Group*Time | 6.89; .002 |  |  |  |

Note: In the *Statistics* column, *F*-ratios were derived from repeated measures analysis of variance (ANOVA) models. In the *Pre-intervention*, *Middle*, and *Post-intervention* columns, *F*-ratios were derived from univariate ANOVA models. *M* = mean. *SD* = standard deviation. PSS = Perceived Stress Scale. CES-D = Center for Epidemiologic Studies Depression Scale. SES = Self-Efficacy Scale. STAI trait = Trait anxiety of State-trait Anxiety Inventory.

**Supplemental Table 9. Impact of personality trait focused digital intervention adjusting for mental health service use during intervention period – Jumpy program**

|  |  | Statistics  *F;* *p-*value | Pre-intervention  (baseline)  *M* ± *SD* | Middle  (Week 5)  *M* ± *SD* | Post-intervention  (Week 10)  *M* ± *SD* |
| --- | --- | --- | --- | --- | --- |
| BAI | Time | 11.32; < .001 |  |  |  |
|  | Mental health service | .19; .66 |  |  |  |
|  | Group | 1.77; .19 | *F* = .45; *P* = .50 | *F* = 2.17; *P* = .15 | *F* = 6.58; *P* = .01 |
|  | Intervention |  | 34.79 ± 12.39 | 23 ± 12.13 | 20.11 ± 13.38 |
|  | Waitlist |  | 31.63 ± 11.34 | 28.21 ± 12.39 | 30 ± 13.90 |
|  | Mental health service *Time | .25; .76 |  |  |  |
|  | Group*Time | 9.07; < .001 |  |  |  |
| PSS | Time | 16.02; < .001 |  |  |  |
|  | Mental health service | .01; .92 |  |  |  |
|  | Group | 7.84; .01 | *F* = .37; *P* = .55 | *F* = 9.86; *P* = .003 | *F* = 9.94; *P* = .003 |
|  | Intervention |  | 26.55 ± 5.15 | 19.71 ± 5.37 | 19 ± 6.29 |
|  | Waitlist |  | 27.37 ± 5.78 | 24.95 ± 5.85 | 24.84 ± 6.32 |
|  | Mental health service *Time | .12; .88 |  |  |  |
|  | Group*Time | 5.48; .006 |  |  |  |
| SES | Time | 2.91; .07 |  |  |  |
|  | Mental health service | .01; .93 |  |  |  |
|  | Group | .86; .36 | *F* = <.01; *P* = .97 | *F* = .80; *P* = .38 | *F* = 2.92; *P* = .09 |
|  | Intervention |  | 61.66 ± 14.71 | 69.08 ± 13.19 | 71.89 ± 13.39 |
|  | Waitlist |  | 62.47 ± 16.9 | 65.16 ± 16.98 | 63.63 ± 16.95 |
|  | Mental health service *Time | 1.16; .31 |  |  |  |
|  | Group*Time | 2.64; .09 |  |  |  |
| CES-D | Time | 5.21; .01 |  |  |  |
|  | Mental health service | .15; .70 |  |  |  |
|  | Group | 1.87; .18 | *F* = .62; *P* = .43 | *F* = 3.05; *P* = .09 | *F* = 8.41; *P* = .005 |
|  | Intervention |  | 60.53 ± 9.97 | 53.58 ± 11.38 | 50.76 ± 12.61 |
|  | Waitlist |  | 61.53 ± 11.07 | 59.26 ± 11.69 | 61.47 ± 10.82 |
|  | Mental health service *Time | .34; .68 |  |  |  |
|  | Group*Time | 2.58; .09 |  |  |  |
| STAI trait | Time | 2.50; .09 |  |  |  |
|  | Mental health service | .24; .62 |  |  |  |
|  | Group | 4.46; .04 | *F* = .02; *P* = .88 | *F* = 4.32; *P* = .04 | *F* = 6.77; *P* = .01 |
|  | Intervention |  | 53.79 ± 11.17 | 47.74 ± 10.57 | 44.32 ± 11.14 |
|  | Waitlist |  | 55.75 ± 11.81 | 54.95 ± 11.97 | 53.70 ± 10.98 |
|  | Mental health service *Time | 2.36; .11 |  |  |  |
|  | Group*Time | 4.38; .02 |  |  |  |

Note: In the *Statistics* column, *F*-ratios were derived from repeated measures analysis of covariance (ANCOVA) models which included mental health service use during intervention period as a covariate. In the *Pre-intervention*, *Middle*, and *Post-intervention* columns, *F*-ratios were derived from univariate ANCOVA models. *M* = mean. *SD* = standard deviation. BAI = Beck Anxiety Inventory. PSS = Perceived Stress Scale. CES-D = Center for Epidemiologic Studies Depression Scale. SES = Self-Efficacy Scale. STAI trait = Trait anxiety of State-trait Anxiety Inventory.

**Supplemental Table 10. Maintenance of intervention effects**

| Total program  *N*=88 | Post-intervention  *M*±*SD* | Follow-up  *M*±*SD* | *t* | *p* |
| --- | --- | --- | --- | --- |
| *Primary outcome measure* |  |  |  |  |
| PSS | 18.26±5.55 | 18.64±6.68 | -.68 | .50 |
| *Secondary outcome measures* |  |  |  |  |
| SES | 72.63±14.16 | 72.92±14.94 | -.37 | .71 |
| CES-D | 19.93±11.88 | 19.89±11.88 | .05 | .96 |
| STAI trait | 46.05±11.61 | 47.08±12.29 | .18 | .86 |
| Riggy program  *N*=21 | Post-intervention  *M*±*SD* | Follow-up  *M*±*SD* | *t* | *P* |
| *Primary outcome measure* |  |  |  |  |
| HMPS | 186.67±28.84 | 186.00±26.77 | .16 | .88 |
| *Secondary outcome measures* |  |  |  |  |
| PSS | 16.86±5.25 | 18.00±5.72 | -1.07 | .30 |
| SES | 80.95±14.04 | 80.43±16.13 | .47 | .65 |
| CES-D | 14.19±9.05 | 15.10±8.62 | -.70 | .49 |
| STAI trait | 45±9.84 | 44.95±10.78 | .03 | .98 |
| Pleaser program  *N*=17 | Post-intervention  *M*±*SD* | Follow-up  *M*±*SD* | *t* | *P* |
| *Primary outcome measure* |  |  |  |  |
| SSES | 58.24±10.44 | 57.06±12.32 | .47 | .64 |
| *Secondary outcome measures* |  |  |  |  |
| PSS | 21.06±5.25 | 21.29±7.59 | -.14 | .89 |
| SES | 67.24±10.56 | 67.24±12.70 | .00 | 1 |
| CES-D | 21.12±9.06 | 21.29±9.12 | -.08 | .94 |
| STAI trait | 50.94±11.22 | 52.35±9.68 | 1.17 | .26 |
| Shelly Program  *N*=24 | Post-intervention  *M*±*SD* | Follow-up  *M*±*SD* | *t* | *P* |
| *Primary outcome measure* |  |  |  |  |
| UCLA LS | 49.54±10.07 | 49.75±12.64 | -.18 | .86 |
| *Secondary outcome measures* |  |  |  |  |
| PSS | 17.08±5.20 | 18.75±6.62 | -1.44 | .16 |
| SES | 70.46±15.37 | 71.92±14.68 | -.95 | .35 |
| CES-D | 17.71±8.83 | 20.67±10.36 | -1.84 | .08 |
| STAI trait | 47.29±9.59 | 48.38±9.64 | -.56 | .58 |
| Jumpy program  *N*=26 | Post-intervention  *M*±*SD* | Follow-up  *M*±*SD* | *t* | *P* |
| *Primary outcome measure* |  |  |  |  |
| BAI | 19.00±13.06 | 19.50±15.60 | -.25 | .81 |
| *Secondary outcome measures* |  |  |  |  |
| PSS | 18.65±5.84 | 17.31±6.92 | 2.37 | .03 |
| SES | 71.42±12.93 | 71.50±13.97 | -.04 | .97 |
| CES-D | 25.85±15.21 | 22.12±13.20 | 1.94 | .06 |
| STAI trait | 50.77±13.01 | 50.46±12.77 | .20 | .84 |

Note. PSS = Perceived Stress Scale. SES = Self-Efficacy Scale. CES-D = Center for Epidemiologic Studies Depression Scale. STAI trait = Trait anxiety of State-trait Anxiety Inventory. HMPS = Hewitt Multidimensional Perfectionism Scale. SSES = State Self-Esteem Scale. UCLA-LS = UCLA Loneliness Scale. BAI = Beck Anxiety Index.

|  | 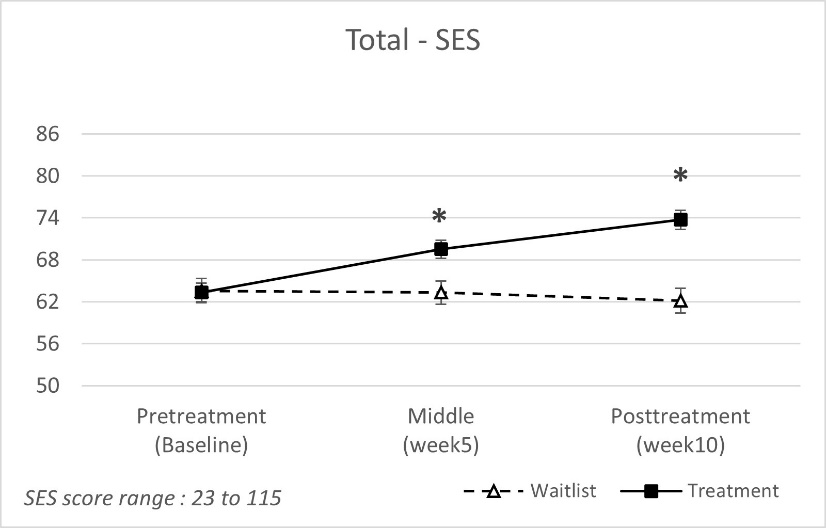 |
| --- | --- |
| 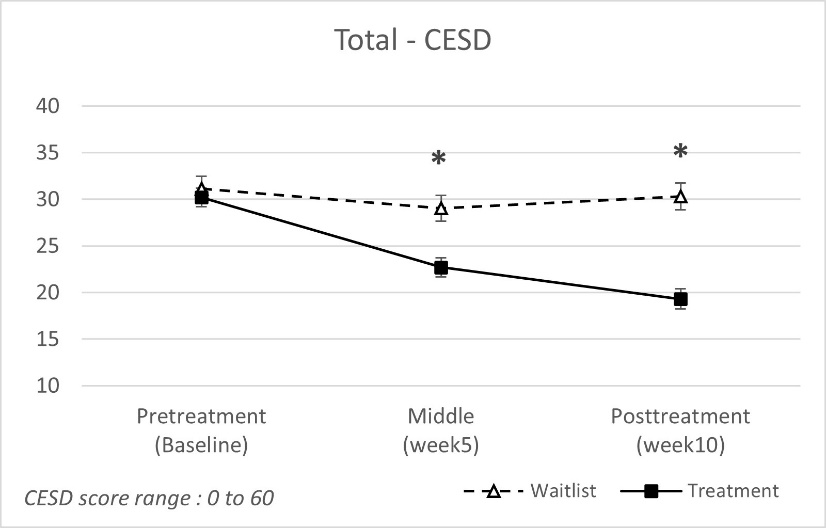 | **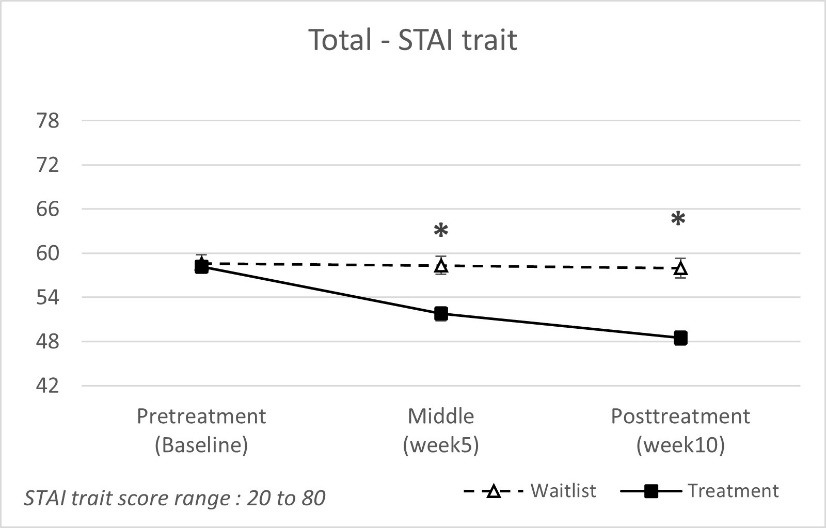** |

**Supplemental Figure 1. Secondary results at each assessment point – Total program**

Note. Means and 95% confidence intervals of the sample mean standard errors. * represents significant group differences in mean outcome scores as per univariate ANOVA. PSS=Perceived Stress Scale. SES = Self-Efficacy Scale. CES-D = Center for Epidemiologic Studies Depression Scale. STAI trait = Trait anxiety of State-trait Anxiety Inventory.

| 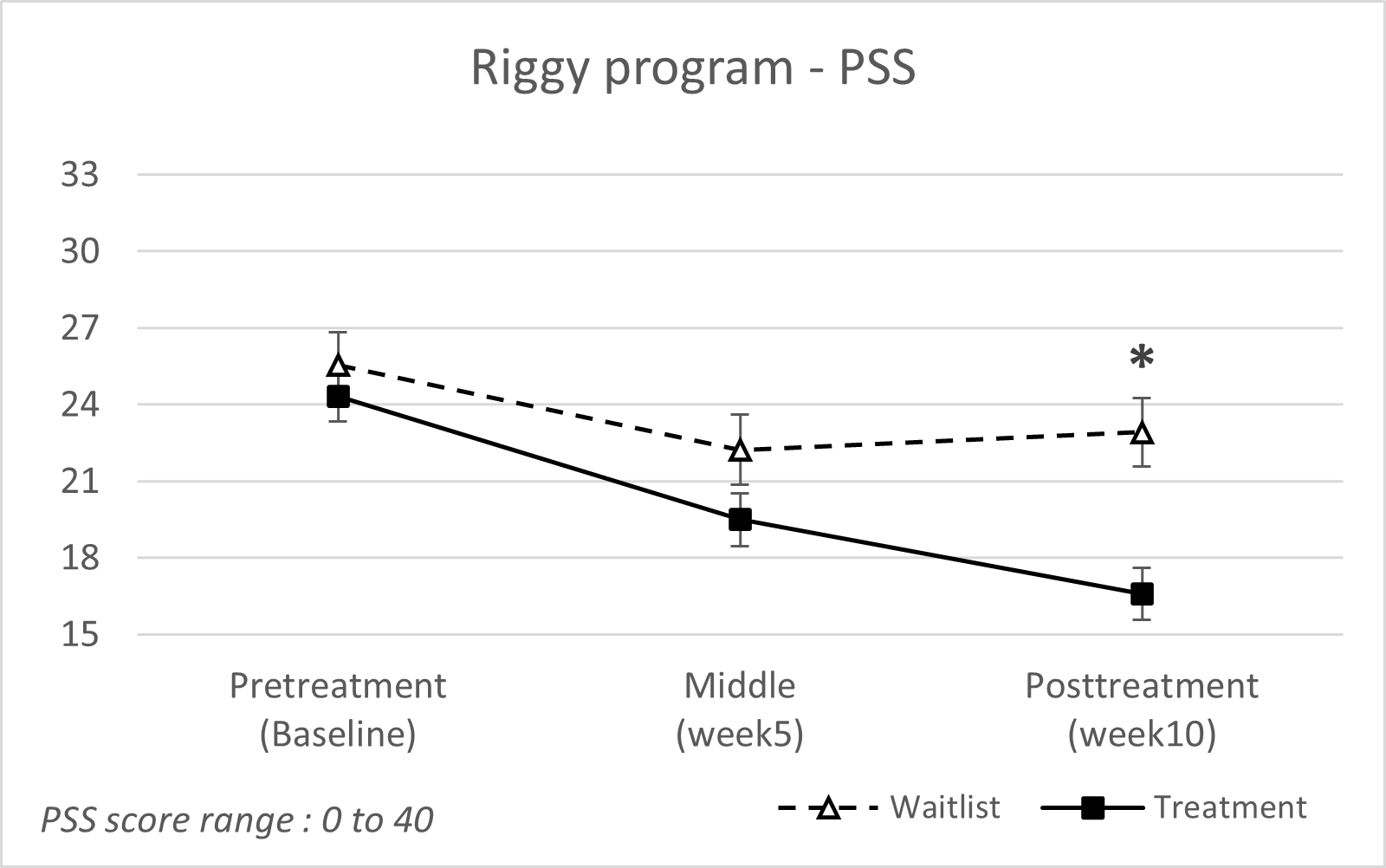 | 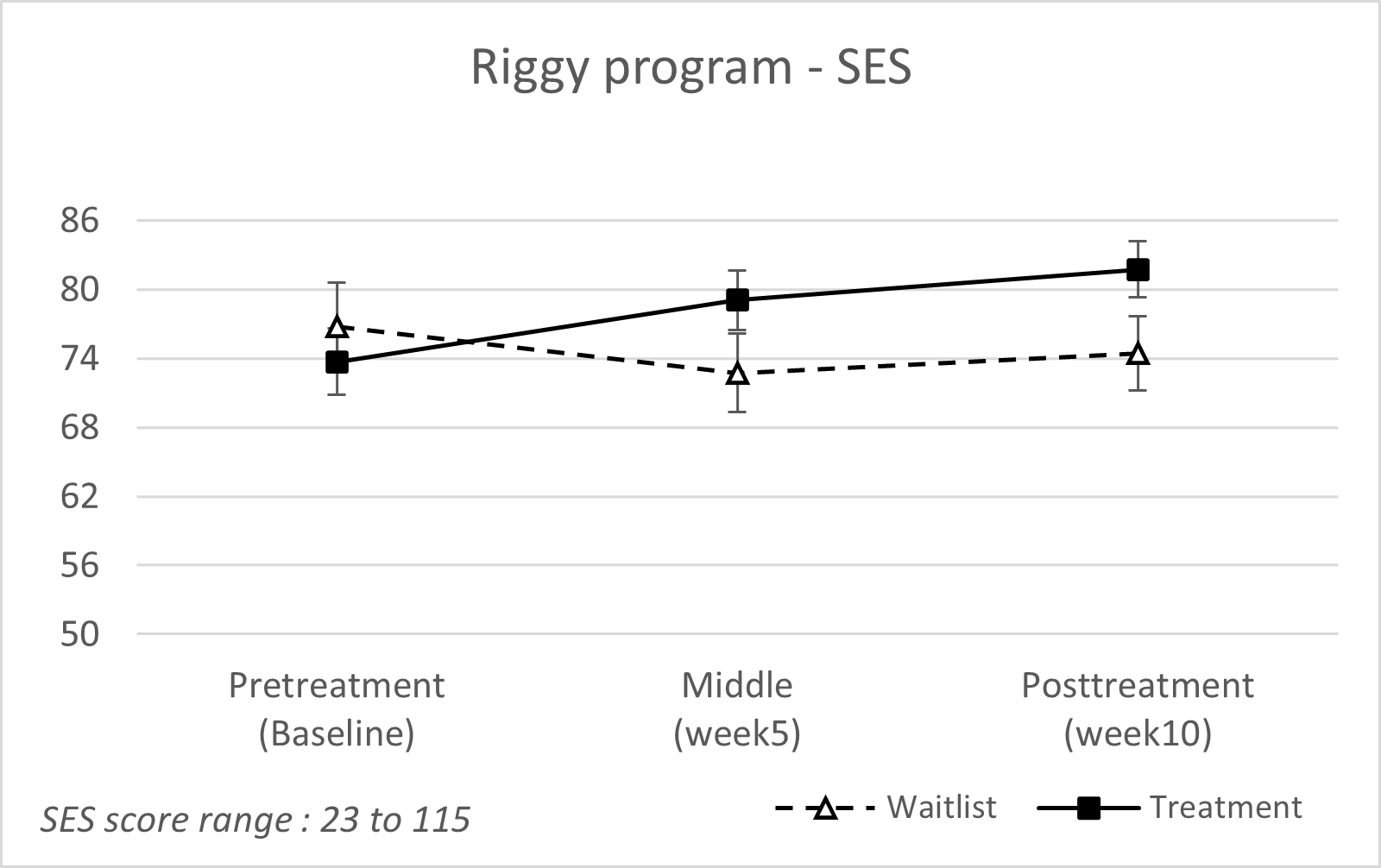 |
| --- | --- |
| 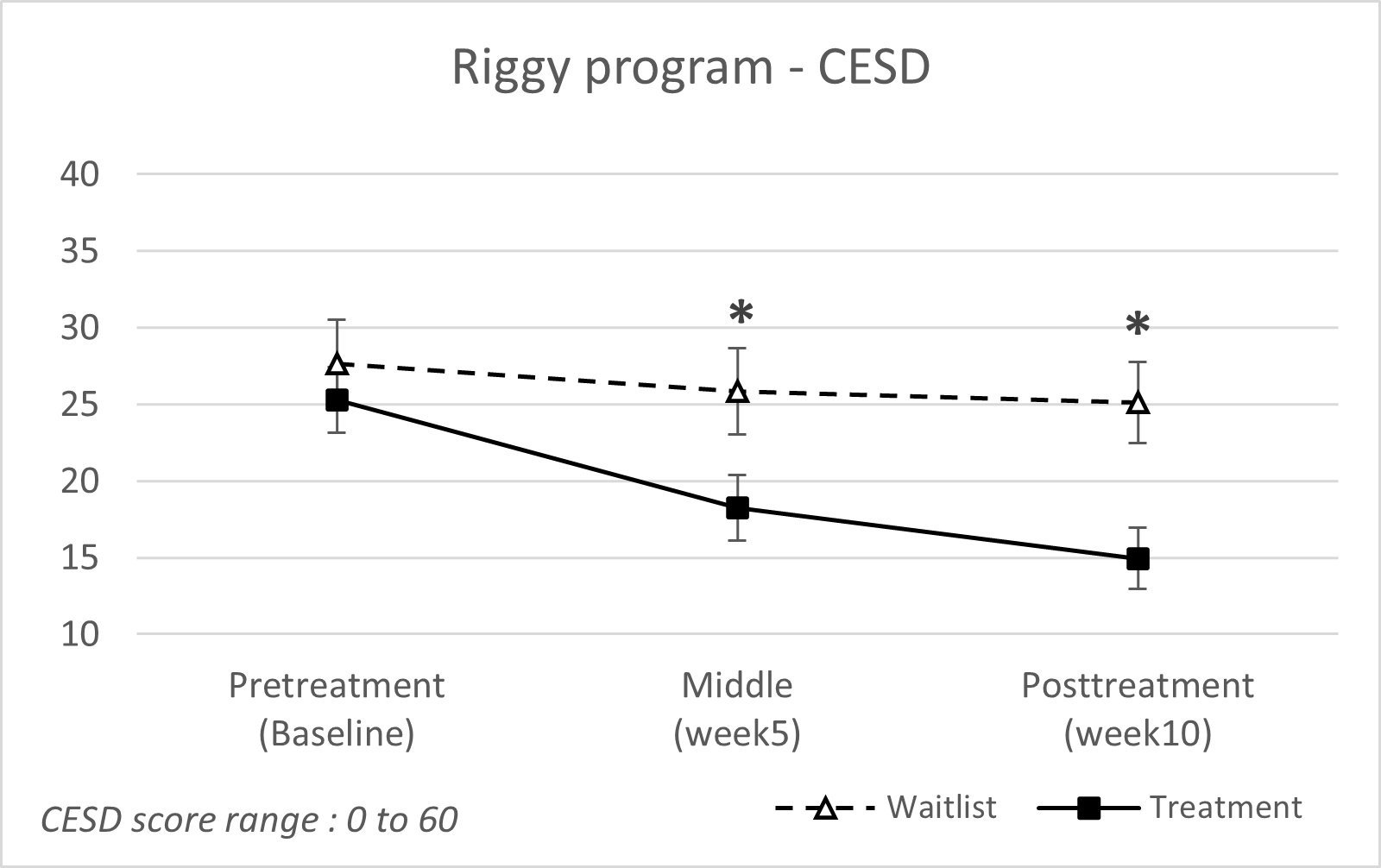 | **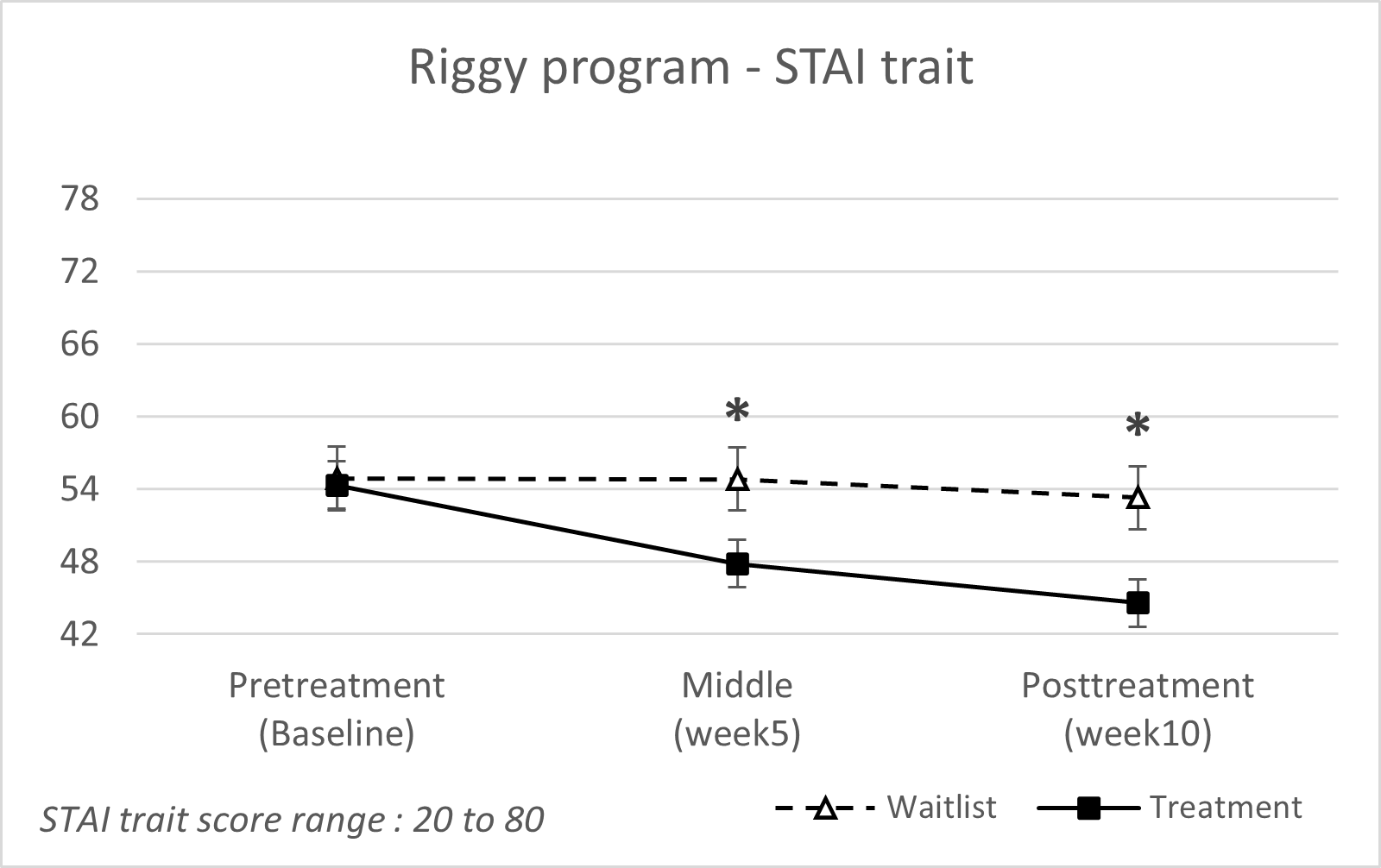** |

**Supplemental Figure 2. Secondary results at each assessment point – Riggy program**

Note. Means and 95% confidence intervals of the sample mean standard errors. * represents significant group differences in mean outcome scores as per univariate ANCOVA. The gray background indicates that the intervention x time interaction is not significant. PSS=Perceived Stress Scale. SES = Self-Efficacy Scale. CES-D = Center for Epidemiologic Studies Depression Scale. STAI trait = Trait anxiety of State-trait Anxiety Inventory.

| 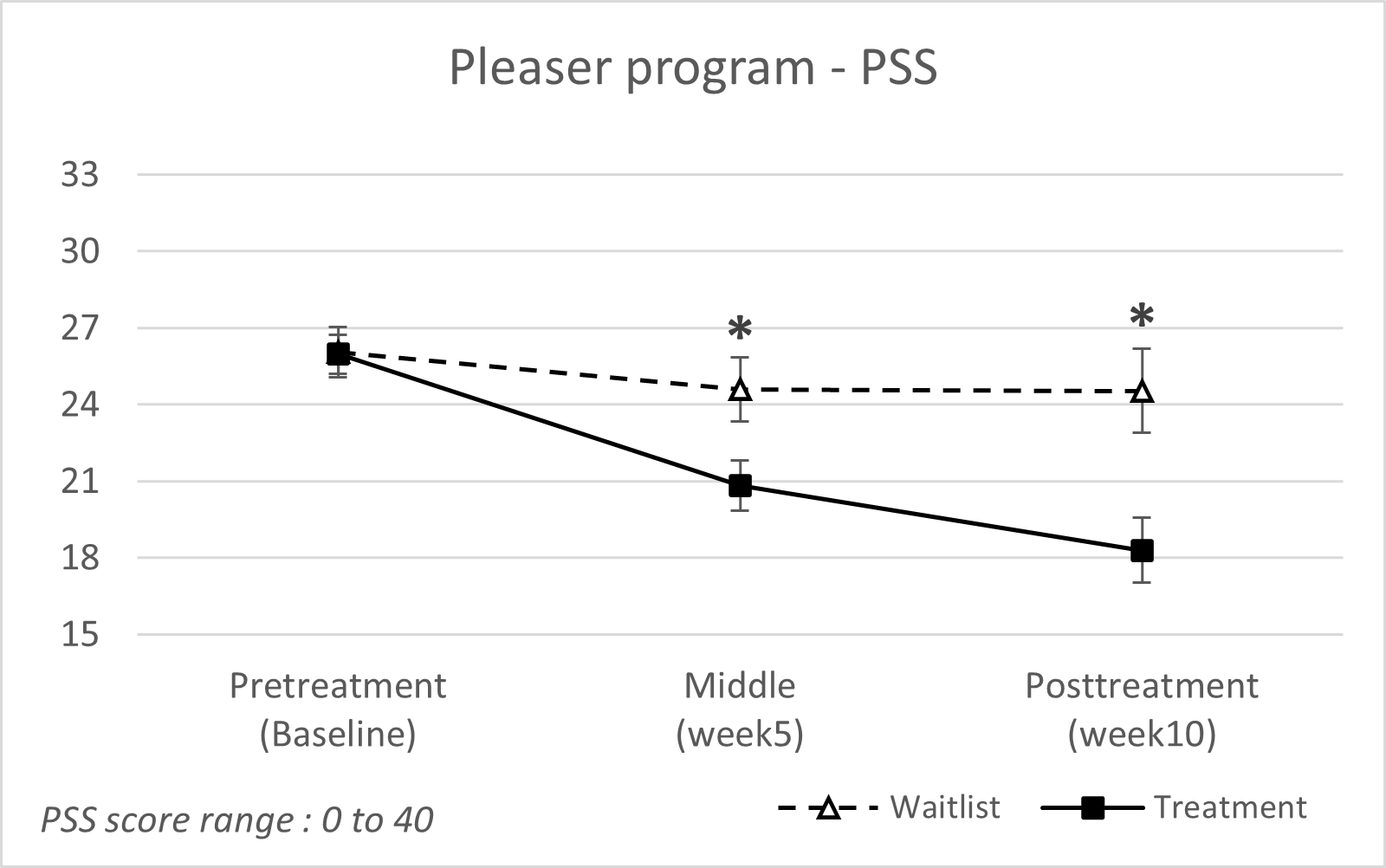 | 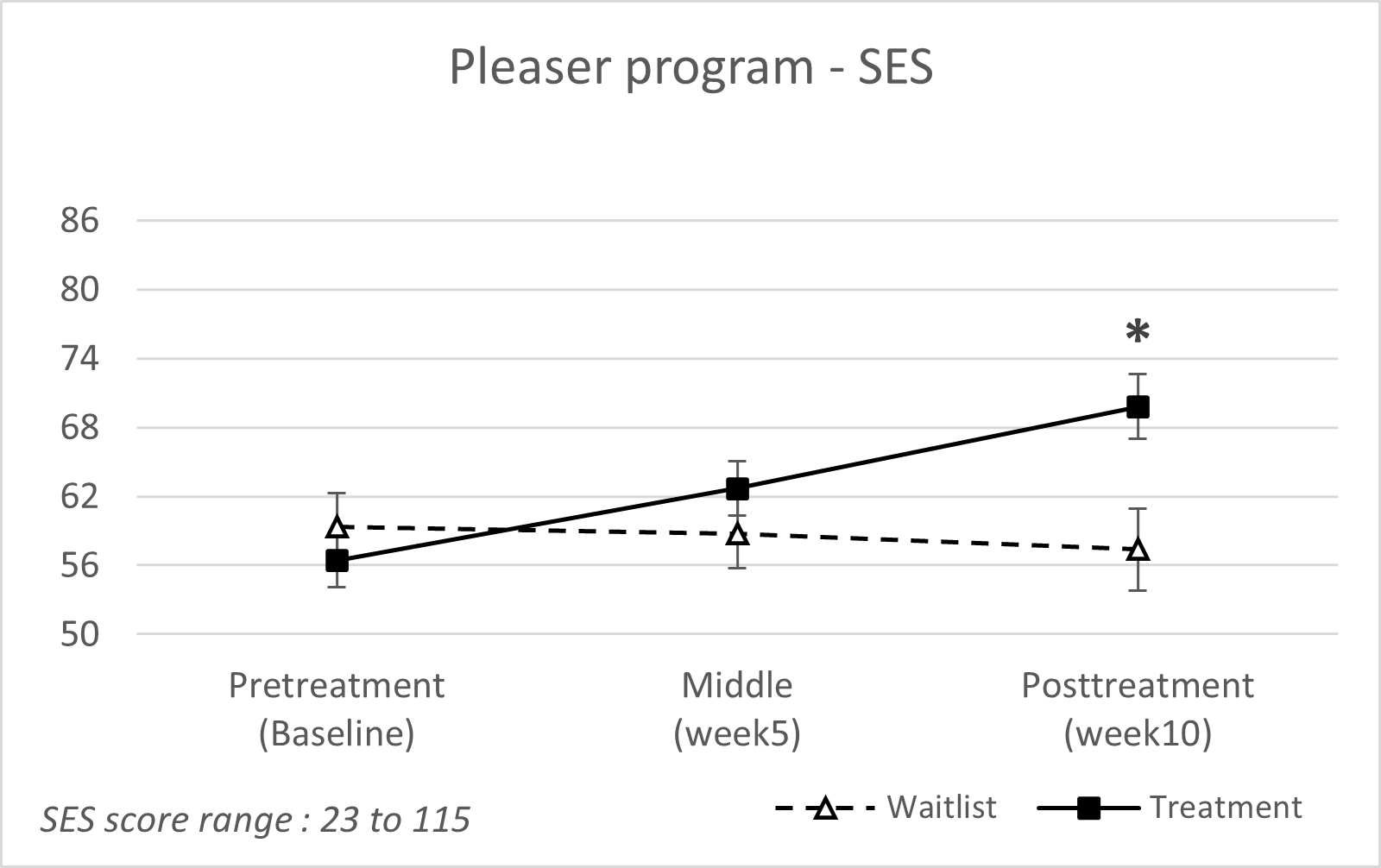 |
| --- | --- |
| 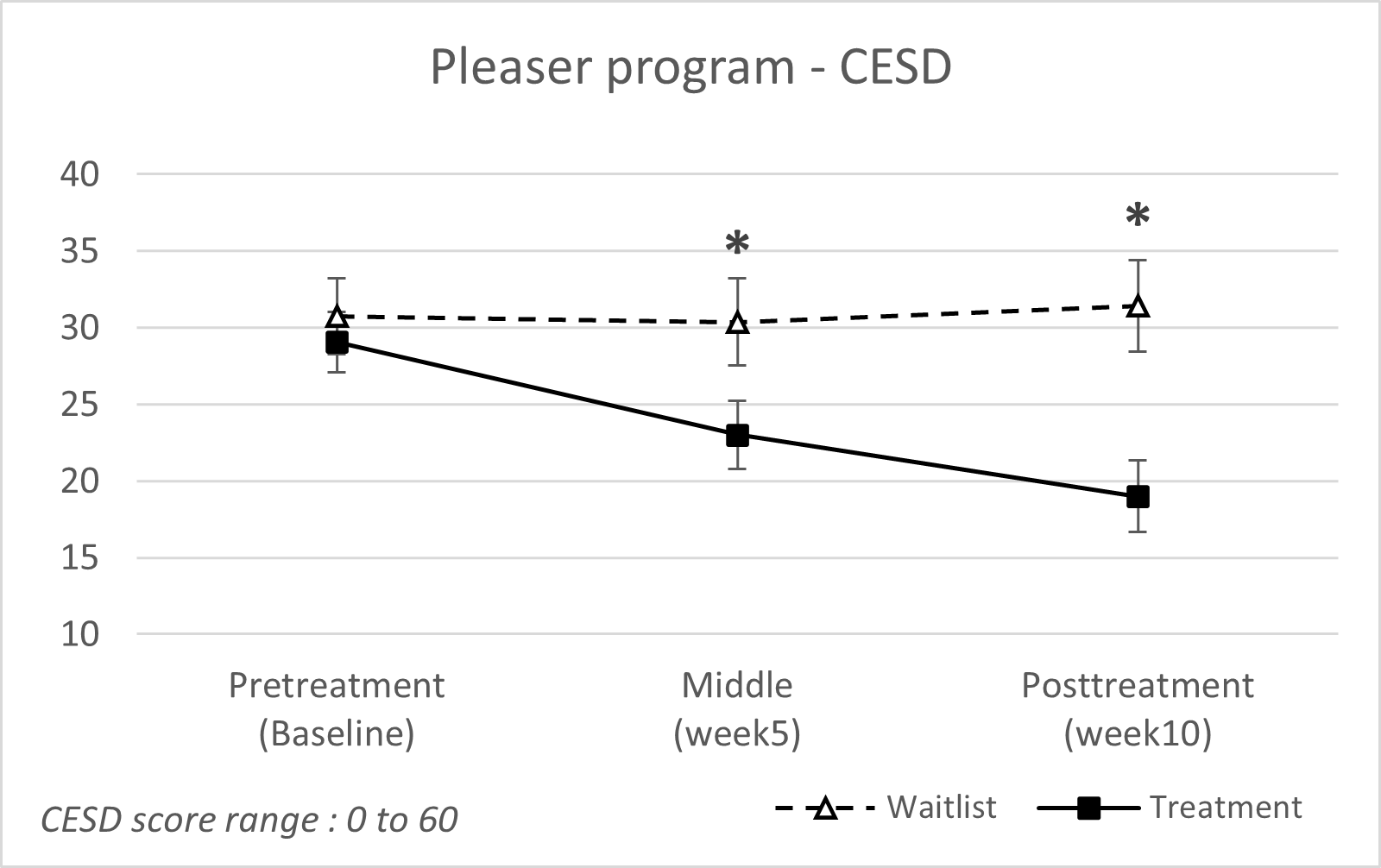 | **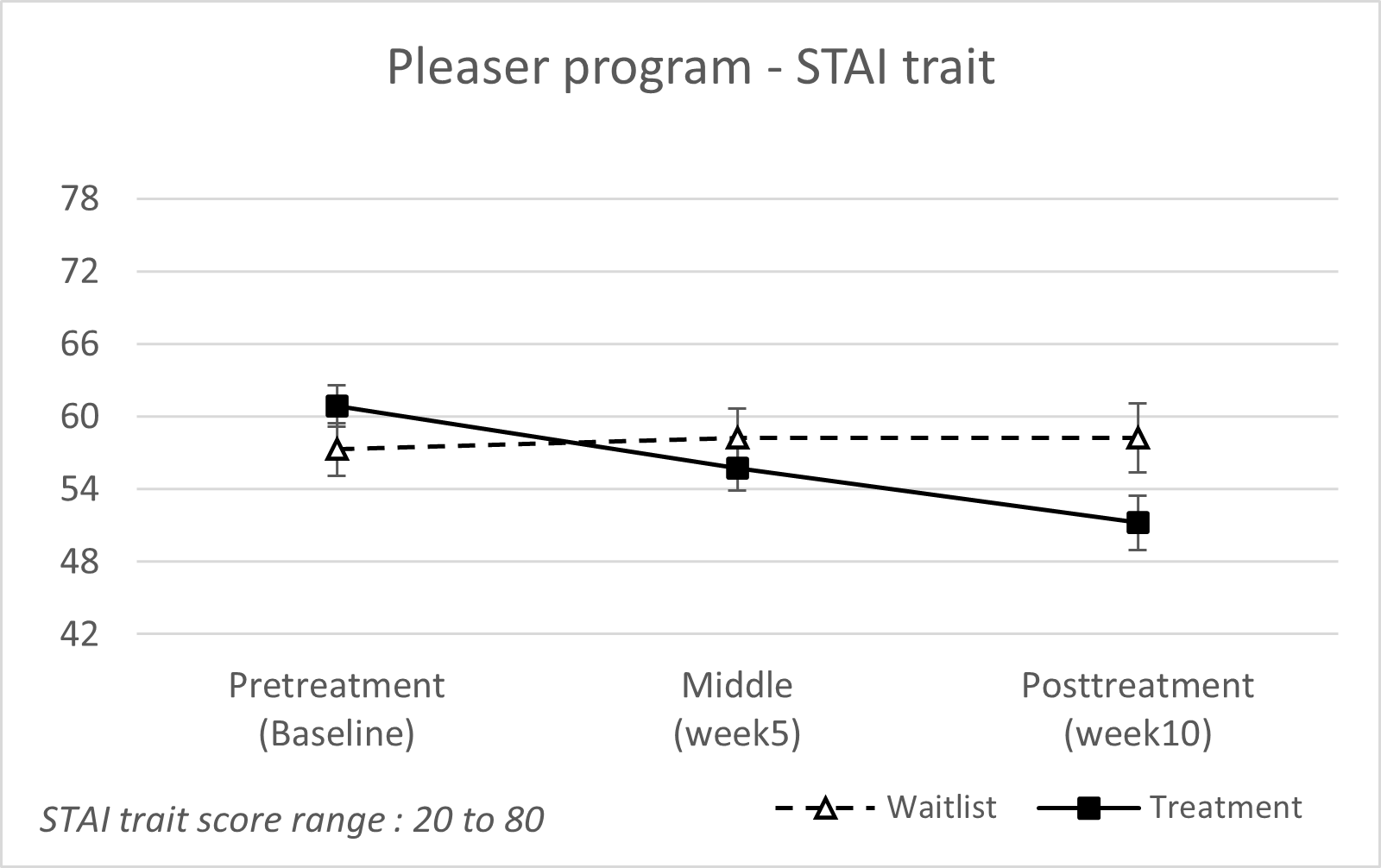** |

**Supplemental Figure 3. Secondary results at each assessment point – Pleaser program**

Note. Means and 95% confidence intervals of the sample mean standard errors. * represents significant group differences in mean outcome scores as per univariate ANOVA. PSS=Perceived Stress Scale. SES = Self-Efficacy Scale. CES-D = Center for Epidemiologic Studies Depression Scale. STAI trait = Trait anxiety of State-trait Anxiety Inventory.

| 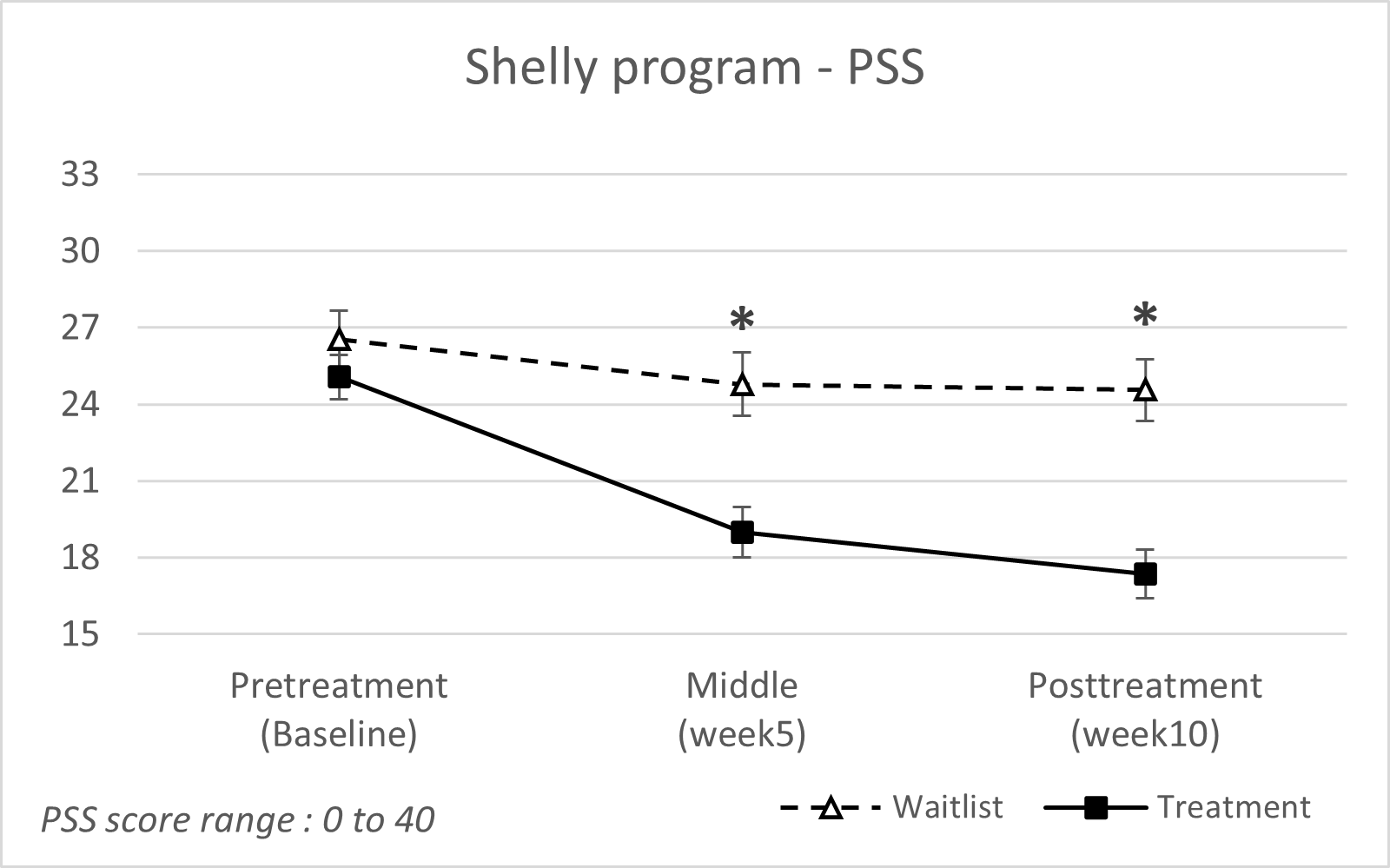 | 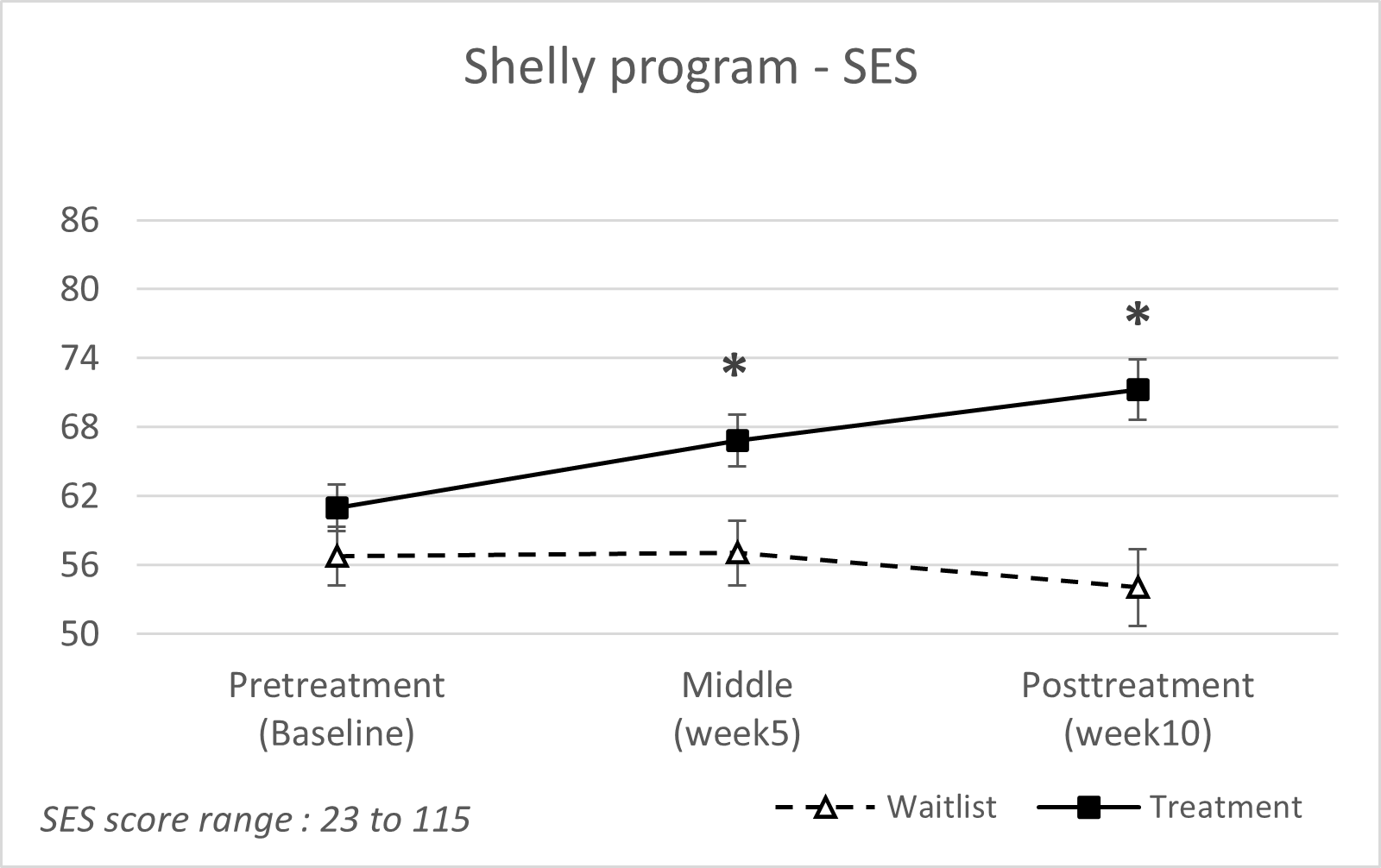 |
| --- | --- |
| 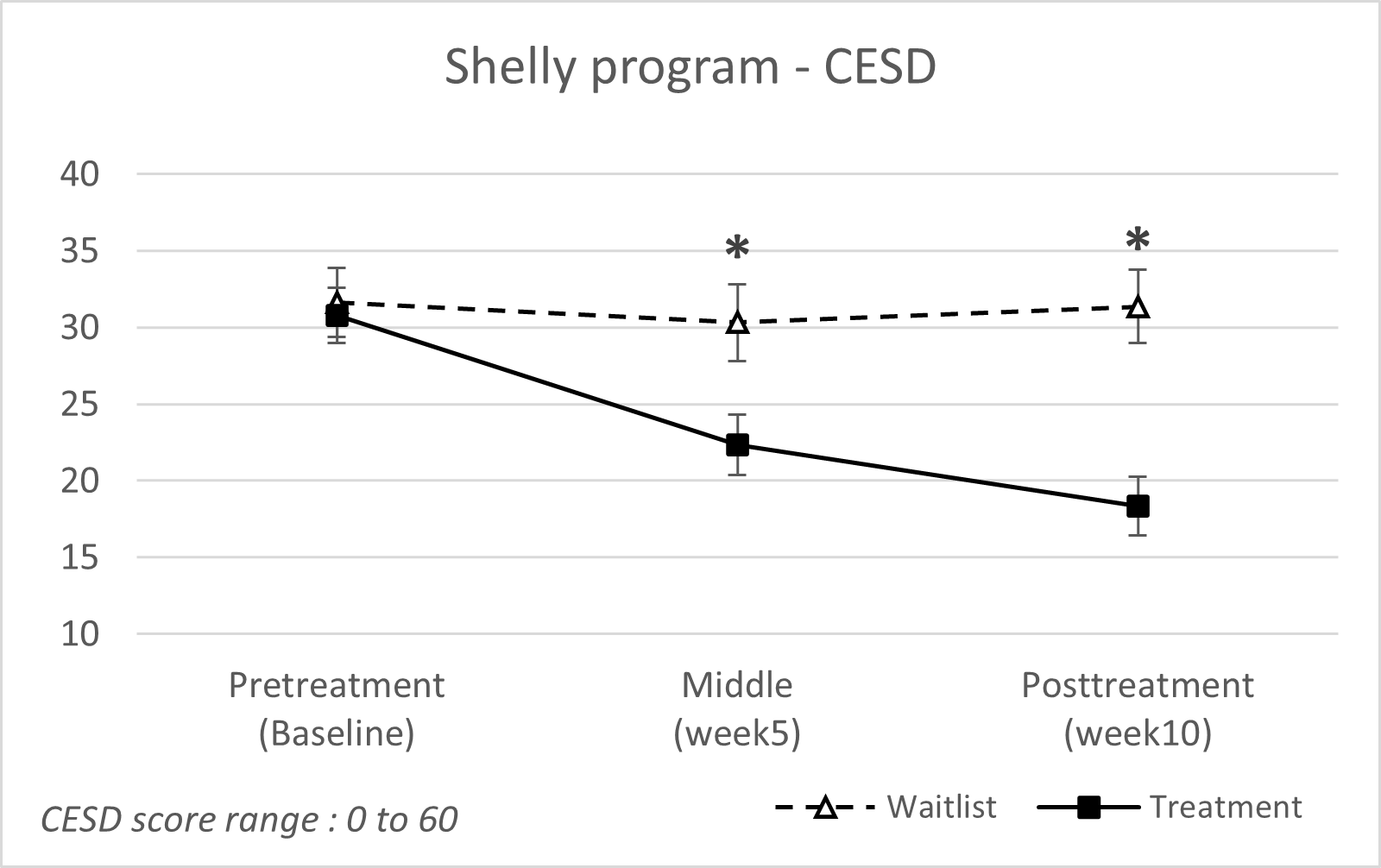 | **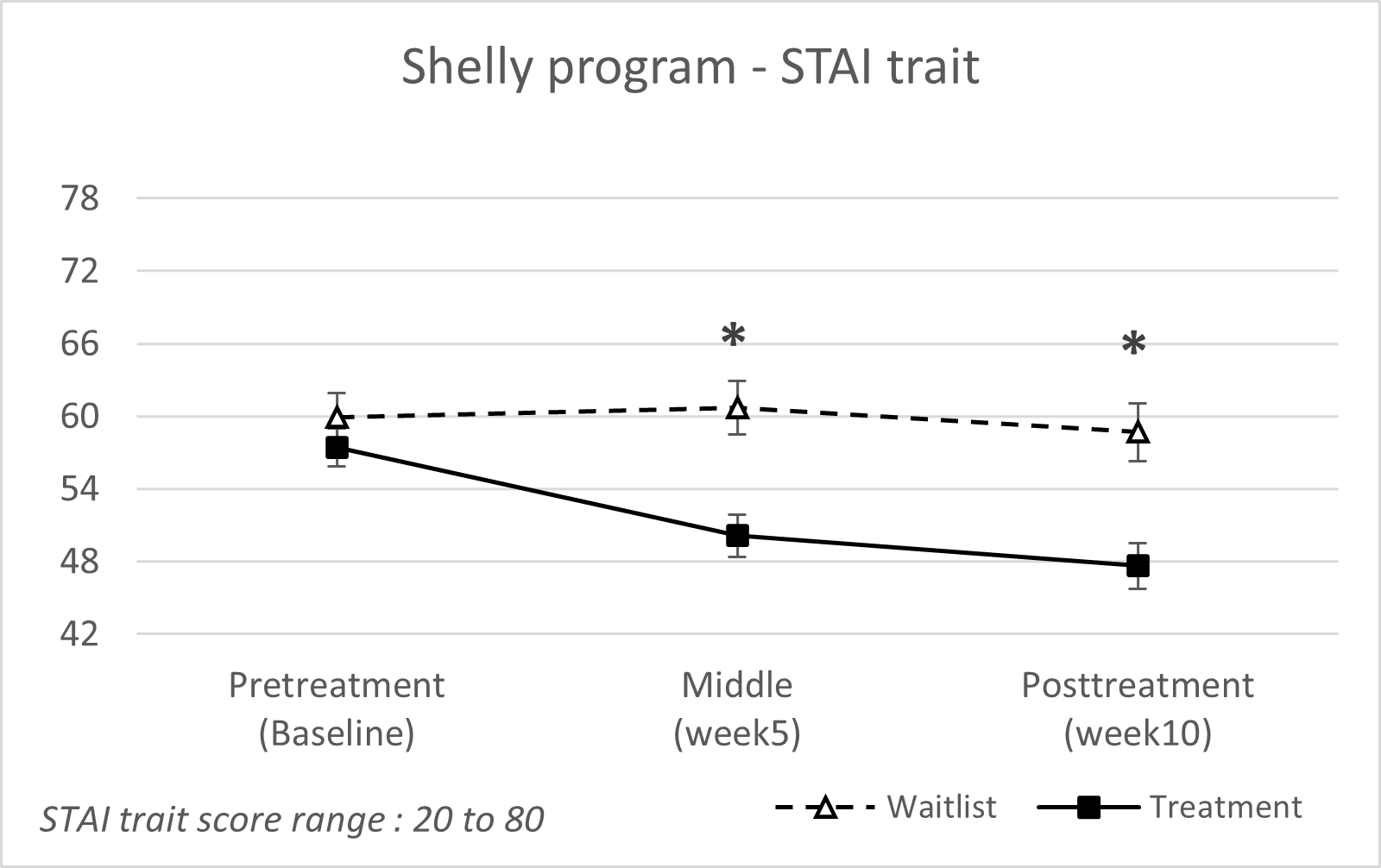** |

**Supplemental Figure 4. Secondary results at each assessment point – Shelly program**

Note. Means and 95% confidence intervals of the sample mean standard errors. * represents significant group differences in mean outcome scores as per univariate ANOVA. PSS=Perceived Stress Scale. SES = Self-Efficacy Scale. CES-D = Center for Epidemiologic Studies Depression Scale. STAI trait = Trait anxiety of State-trait Anxiety Inventory.

| 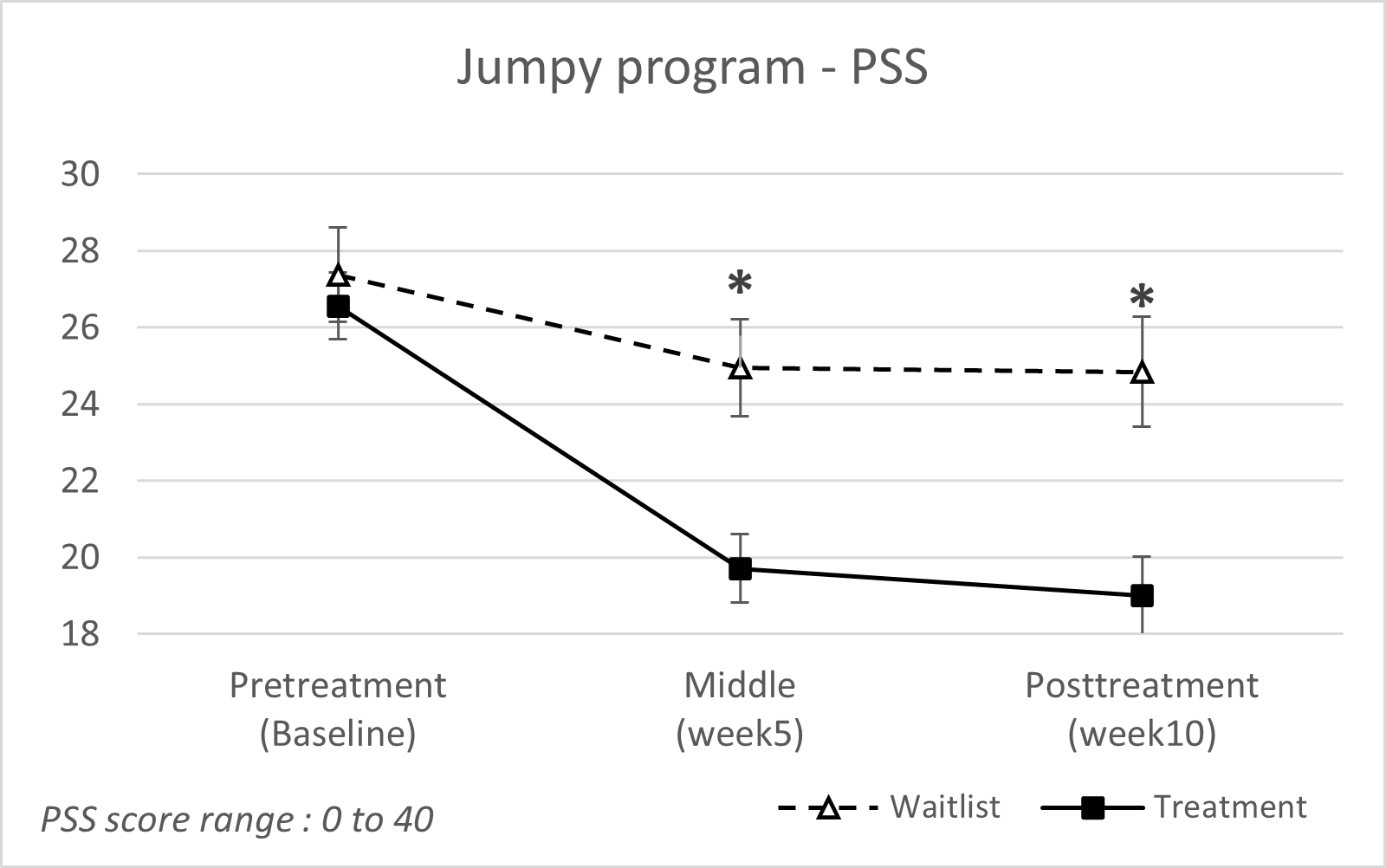 | 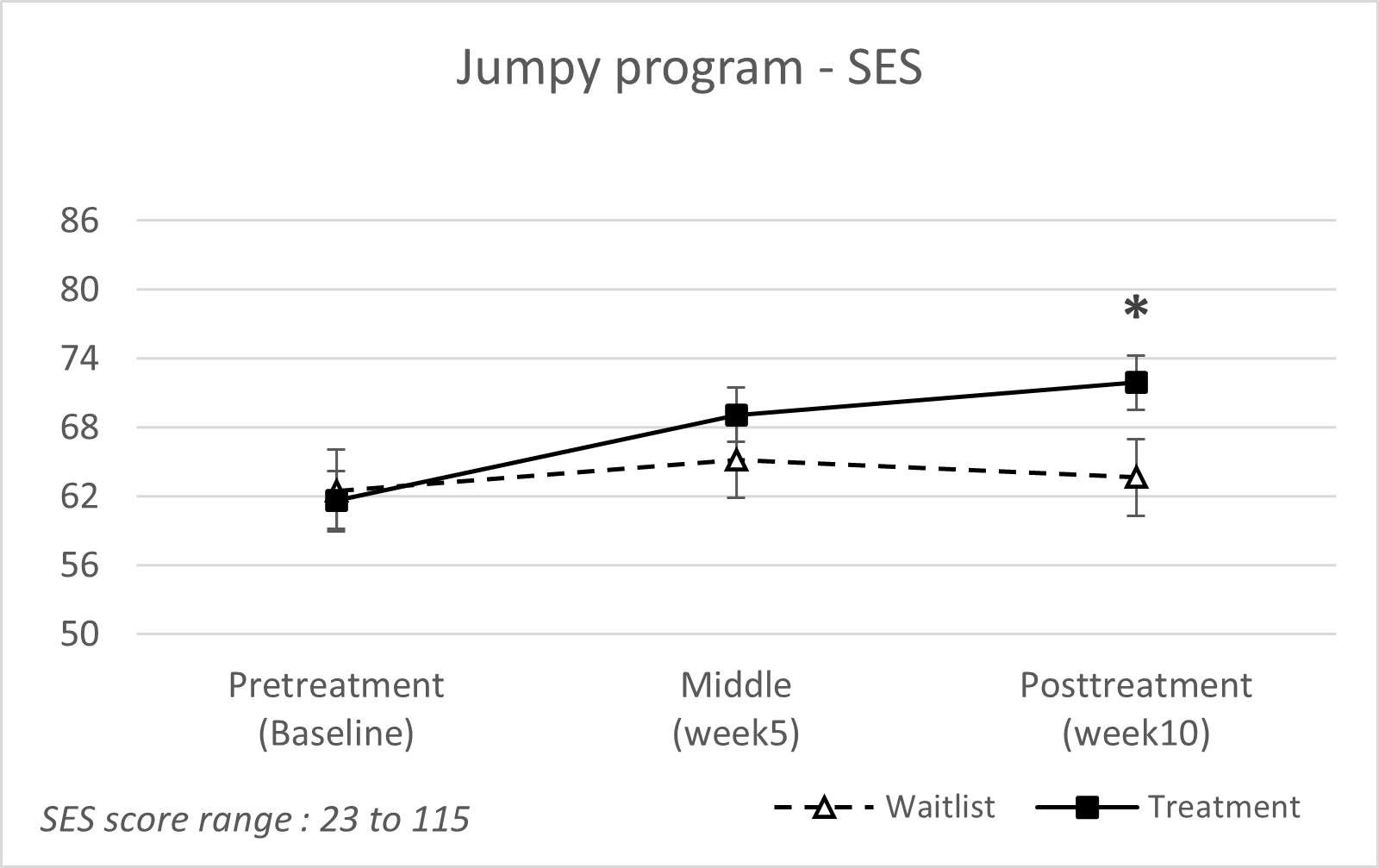 |
| --- | --- |
| 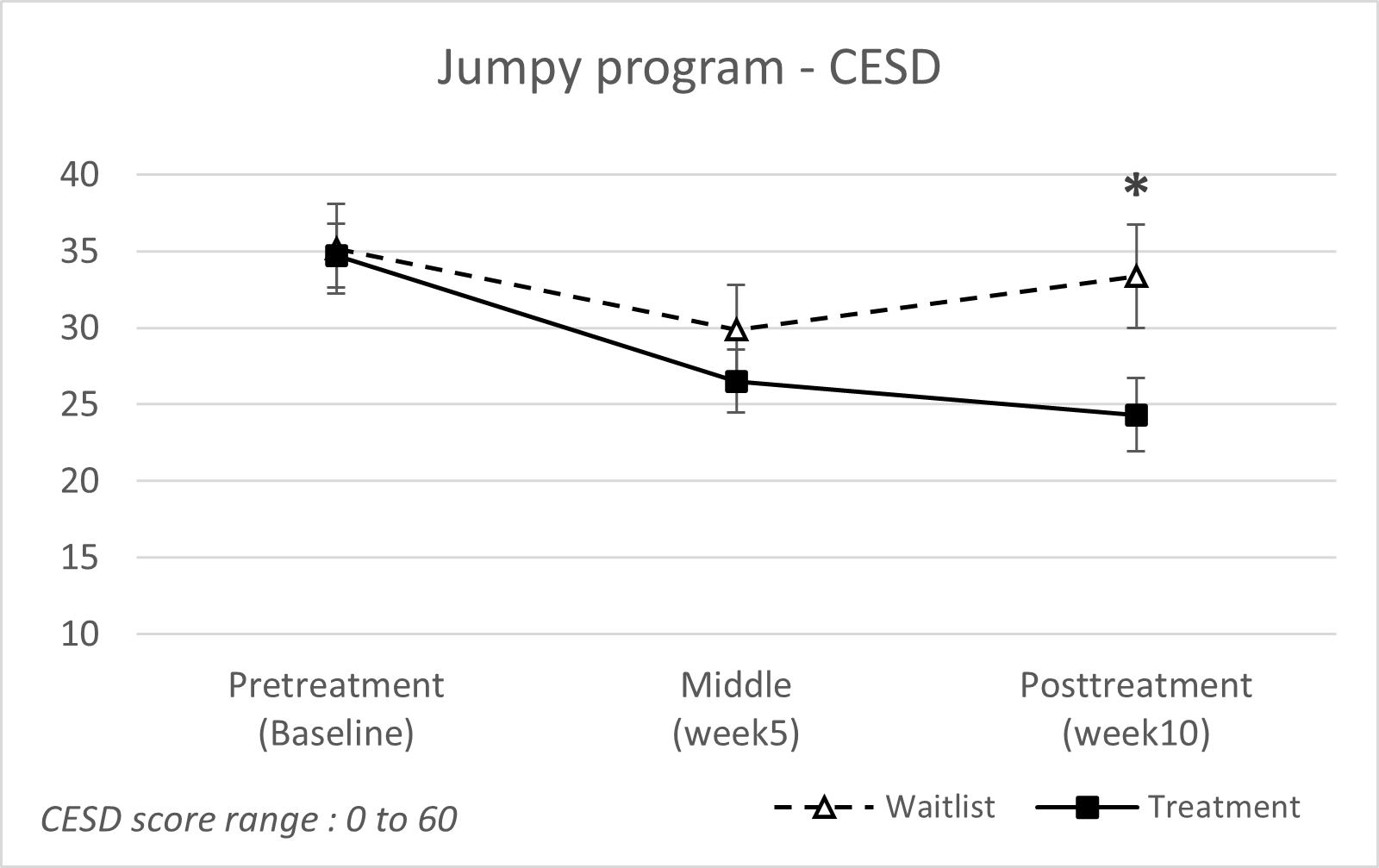 | **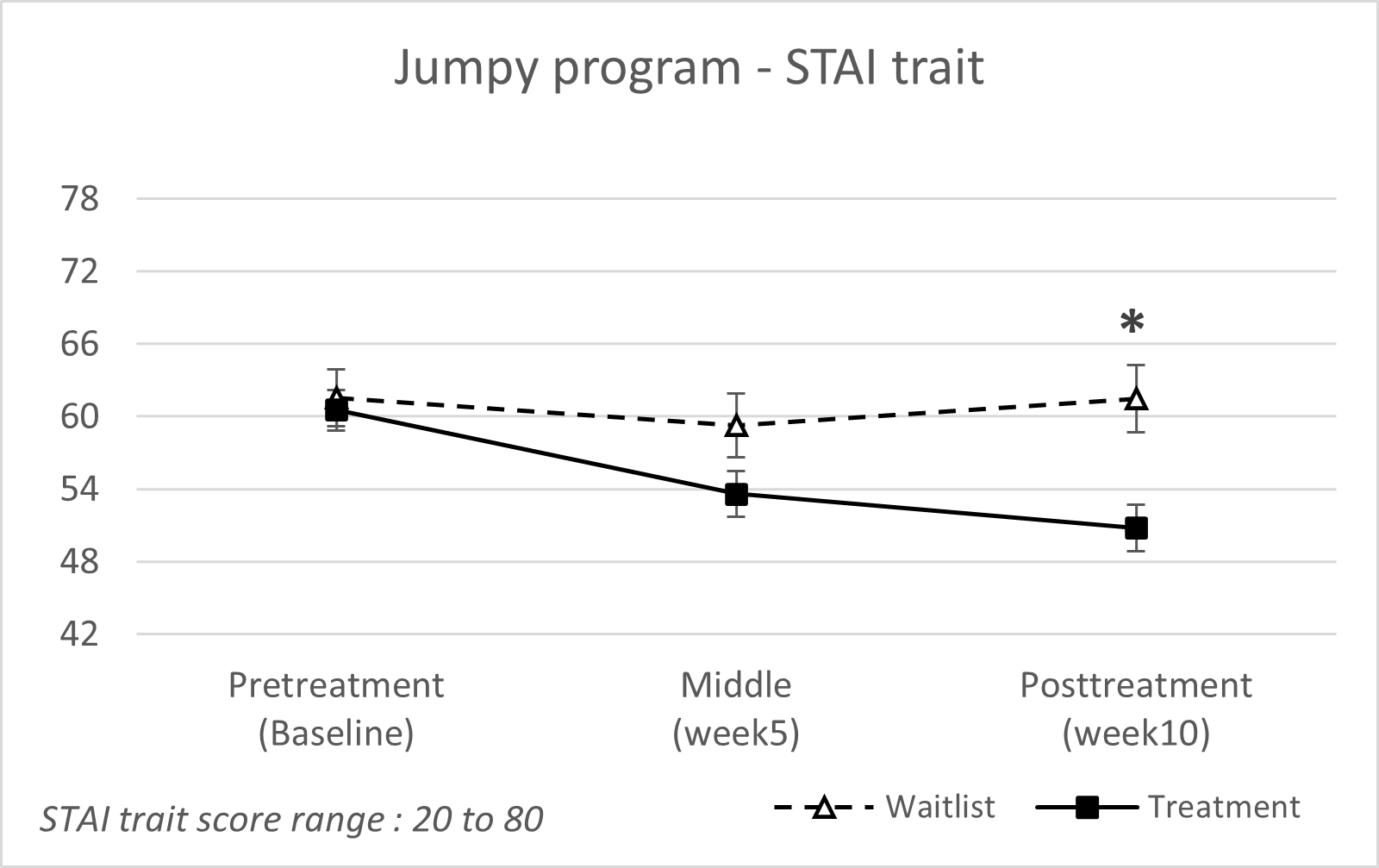** |

**Supplemental Figure 5. Secondary results at each assessment point – Jumpy program**

Note. Means and 95% confidence intervals of the sample mean standard errors. * represents significant group differences in mean outcome scores as per univariate ANOVA. PSS=Perceived Stress Scale. SES = Self-Efficacy Scale. CES-D = Center for Epidemiologic Studies Depression Scale. STAI trait = Trait anxiety of State-trait Anxiety Inventory.
